# Loss of exosomal miR-146a-5p from cancer-associated fibroblasts after androgen deprivation therapy contributes to prostate cancer metastasis

**DOI:** 10.1186/s13046-020-01761-1

**Published:** 2020-12-14

**Authors:** Yu Zhang, Jing Zhao, Mao Ding, Yiming Su, Di Cui, Chenyi Jiang, Sheng Zhao, Gaozhen Jia, Xiaohai Wang, Yuan Ruan, Yifeng Jing, Shujie Xia, Bangmin Han

**Affiliations:** 1 Department of Urology, Shanghai General Hospital, Shanghai Jiao Tong University School of Medicine, Wujin Road 85, Shanghai, 200080 China; 2 Institute of Urology, Shanghai Jiao Tong University, Shanghai, 200080 China

**Keywords:** Prostate cancer, Androgen deprivation therapy (ADT), Metastasis, Cancer-associated fibroblasts (CAFs), Exosomes

## Abstract

**Background:**

Androgen deprivation therapy (ADT) is the backbone of therapy for advanced prostate cancer (PCa). Despite the good initial response, castration resistance and metastatic progression will inevitably occur. Cancer-associated fibroblasts (CAFs) may be implicated in promoting metastasis of PCa after ADT. Our aim is to investigate the role and mechanism of CAFs-derived exosomes involving in metastasis of PCa after ADT.

**Methods:**

PCa cells were co-cultured with exosomes derived from 10 nM dihydrotestosterone (DHT)-treated (simulating the high androgen level of prostate cancer microenvironment) or ethanol (ETOH) -treated (simulating the castration level of prostate cancer microenvironment after ADT) CAFs, and their migration and invasion differences under castration condition were examined both in vitro and in vivo. The miRNA profiles of exosomes derived from DHT-treated CAFs and matched ETOH-treated CAFs were analysed via next generation sequencing. The transfer of exosomal miR-146a-5p from CAFs to PCa cells was identified by fluorescent microscopy. The function and direct target gene of exosomal miR-146a-5p in PCa cells were confirmed through Transwell assays, luciferase reporter, and western blot.

**Results:**

Compared with DHT-treated CAFs, exosomes derived from ETOH-treated CAFs dramatically increased migration and invasion of PCa cells under castration condition. MiR-146a-5p level in exosomes from ETOH-treated CAFs was significantly reduced. The loss of miR-146a-5p may strengthen the epithelial-mesenchymal transition (EMT) to accelerate cancer cells metastasis by modulating epidermal growth factor receptor (EGFR)/ERK pathway.

**Conclusions:**

CAFs-derived exosomal miR-146a-5p confers metastasis in PCa cells under ADT through the EGFR/ERK pathway and it may present a new treatment for PCa.

## Background

Prostate cancer (PCa) is the most common malignancy and the second leading major of cancer death in American males, with an estimated 191,930 new cases and 33,330 deaths expected in 2020 [1]. Androgen deprivation therapy (ADT), including surgical or chemical castration, is the backbone of treatment for all stages of recurrent prostate cancer [2]. Although highly responsive to this therapy initially, eventually almost all patients will evolve into castration-resistant prostate cancer (CRPC) in 18-24 months [3]. Consequently, the treatment failed, and most patients died from metastasis. Therefore, understanding the underlying mechanisms of PCa cells castration resistance and metastasis after ADT remains an emergent need.

Recent researches revealed that in the microenvironment of prostate cancer, cancer-associated fibroblasts (CAFs) are among the most crucial components and are reported to promote tumorigenesis and progression [4–6]. In addition, several studies demonstrated that CAFs could promote androgen independence and metastatic progression in prostate cancer [7–9]. Significantly, the effect of inhibition of AR signalling in CAFs on PCa metastasis has attracted great attention. Yu et al. demonstrated that AR in CAFs promoted invasion of PCa cells via regulating a series of growth factors [10]. However, Bianca et al. revealed that inhibiting AR signalling in CAFs could promote prostate cancer cell migration by secreting CCL2 and CXCL8 [11]. Damien et al. described that AR signalling in CAFs inhibits prostate cancer cell invasion via maintaining the extracellular matrix (ECM) [12]. Therefore, the roles of AR in CAFs are still controversial and have been a hot topic in prostate cancer research.

Exosomes are nanovesicles which range in size from 30 to 150 nm [13]. Exosomes can transmit intracellular cargos which include microRNAs (miRNAs), messenger RNAs (mRNAs), long non-coding RNAs (lncRNAs) and proteins to participate in intercellular communication [14, 15]. Recently, exosome-mediated miRNA delivery has drawn much attention and many studies have showed that exosomal microRNAs contribute to cancer development, such as tumour progression, metastasis, and drug resistance [16–18]. In addition to exosomal miRNAs derived from cancer cells, CAFs-derived exosomal miRNAs have also been found to contribute to therapy resistance in various cancers. Qin et al. demonstrated that exosomal miR-196a from CAFs modulate cisplatin resistance in head and neck cancer [19]. In ovarian cancer, miR-21 could be transferred from CAFs to cancer cells via exosomes, which decreases tumour cell apoptosis and promotes paclitaxel resistance by binding to APAF1 [20]. However, whether CAFs-derived exosomes react to ADT are still unclear. Furthermore, the functions of these exosomal miRNAs in regulating metastasis phenotypes of PCa cells have not been clarified.

Herein, we identified that exosomes from CAFs exposed to ADT significantly promoted cell migration and invasion in PCa cells both in vitro and in vivo. By sequencing and verified experiments, we demonstrated that miR-146a-5p was decreased in the exosomes derived from CAFs after ADT. Mechanistically, loss of exosomal miR-146a-5p promoted epithelial-mesenchymal transition (EMT), migration and invasion of PCa cells via activating epidermal growth factor receptor (EGFR)/ERK pathway. Our findings represent a new important molecular mechanism of metastasis in prostate cancer after hormone therapy. Moreover, it suggests a promising therapeutic strategy to suppress prostate cancer metastasis for patients receiving ADT.

## Materials and methods

### Isolation of primary CAFs and cell culture

Tumour tissues were obtained from PCa patients treated by radical prostatectomy at the Department of Urology, Shanghai General Hospital, Shanghai Jiao Tong University, School of Medicine. As previously described [21], primary CAFs were isolated from PCa tissues. In this study, we obtained three sets of primary CAFs from three patients, they did not receive any therapy before radical prostatectomy and their characteristics are shown in Additional file 1: Figure S1. All primary CAFs between passages 2 and 10 were used for all experiments. CAF markers including Vimentin, FAP, and α-SMA were determined by immunofluorescence. Androgen deprived prostate tissues were required in radical prostatectomy from patients treated for a long-term period (3–6 months) with abiraterone and leuprolide.

hTERT PF179T CAF were acquired from American Type Culture Collection (ATCC, VA, USA, Cat# CRL-3290), and HEK 293 T, LNCaP, and DU145 were acquired from Cell Bank of Shanghai Institute of Cells, Chinese Academy of Science (Shanghai, China). The prostate cell lines were authenticated (GENEWIZ, Suzhou, China). Cells were incubated in the suitable medium (RPMI-1640 for LNCaP, DU145; DMEM for CAFs and 293 T) supplemented with 10% fetal bovine serum (FBS), 1% penicillin/streptomycin. All cells were cultured at 37 °C with 5% CO2.

### Plasmid construction, lentivirus packaging, and cell transfection

Lentiviral plasmids encoding miR-146a-5p or negative control and lentivirus were obtained from Genomeditech (Shanghai, China). To create miR-146a-5p overexpressing LNCaP and DU145 cell lines, we transfected cells with lentivirus according to the manufacturer’s instructions and selected stable cell lines with puromycin.

miR-146a-5p mimic and miR-146a-5p negative-control were purified by RiboBio (Guangzhou, China). The sequences of mimics are as follows: miR-146a-5p mimics: sense: 5’UGAGAACUGAAUUCCAUGGGUU3’, antisense: 5’AACCCAUGGAAUUCAGUUCUCA3’. MiR-NC mimics: sense: 5’UUUGUACUACACAAAAGUACUG3’, antisense: 5’CAGUACUUUUGUGUAGUACAAA3’. EFGR overexpression vector and negative-control plasmids were purchased from Youbio (Changsha, China). Transfections of miRNAs or plasmids were carried out using Lipofectamine 3000 transfection reagent (Invitrogen/Thermo Fisher Scientific) following the manufacturer’s protocol.

### Exosomes isolation and characterization

Androgen receptor (AR) signalling in CAFs was activated by adding dihydrotestosterone (DHT, ApexBio Technology, Houston, USA) to 10% charcoal - stripped FBS (CSFBS) DMEM medium at 10 nM [10]. CAFs were pretreated with 10% CSFBS DMEM medium for 24 h in advance, then CAFs were incubated with freshly medium with 10 nM DHT (simulating the high androgen level of prostate cancer microenvironment) or ethanol (simulating the castration level of prostate cancer microenvironment after ADT) for 48 h. After exposure, CAFs were washed using PBS and cultured in complete medium containing exosome-free CSFBS with 10 nM DHT or ethanol (ETOH) for another 48 h. Exosomes were collected from supernatants of primary CAFs and isolated by ultracentrifugation as previously described [22]; however, we made some modifications. Briefly, cell culture supernatants were gathered and centrifuged at 300g for 10 min, 2000 ×g for 10 min and 10,000 ×g for 30 min. Then, we filtered the supernatants through 0.22 μm filters (Millipore, USA) and ultracentrifuged at 100,000 ×g for 70 min at 4 °C [23]. After removing the cell supernatants, we resuspended the pellets with ice-cold PBS. Next, we ultracentrifuged the suspension at 100,000 ×g for another 70 min at 4 °C. Finally, we resuspended exosomes in PBS and stored at −80 °C. We used GW4869 (Sigma Aldrich, St. Louis, USA) to inhibit exosome release at a concentration of 20 μM. We used BCA methods to measure the concentration of exosomes. Exosomes were observed using transmission electron microscopy and identified by the expression of TSG101 and CD81, which are positive exosome markers. We also detected the concentration and hydrodynamic diameter of exosomes through a NanoSight NS300 Nanoparticle Tracking Analyzer (NTA; Malvern Instruments Ltd, UK) equipped with NTA 3·0 analytical software. Nanoparticle Tracking Analyzer is designed to obtain the particle size distribution of the sample in the liquid suspension by using the characteristics of light scattering and Brownian motion. At present, NTA has been recognized as one of the means of determining exosomes characterization in the field of exosomes research [24, 25].

### Immunofluorescence

Cells grown on cover slips were fixed with 4% paraformaldehyde for 15 min at 25 °C, treated with 0.1% Triton X-100 for 5 min at 4 °C, blocked in 5% donkey serum for 2 h at room temperature, and incubated with primary antibodies against α-SMA (Abcam, Cambridge, MA, USA, Cat# ab7817), Vimentin (Abcam, Cat# ab92547), FAP (Abcam, Cat# ab53066) at 4 °C overnight. After that, cells were incubated with an Alexa Fluor 488-conjugated antibodies (Abcam, Cat# ab150109) or an Alexa Fluor 647-conjugated antibodies (Abcam, Cat# ab150075) for 30 min at 25 °C in the dark, and we treated the slips with 4′,6-diamidino-2-phenylindole (DAPI; Invitrogen, USA) to detect cell nuclei. Cells were observed and pictures were taken by a fluorescence microscope (Leica Microsystems, Germany).

### Western blot analysis

Total proteins were prepared, and western blot analysis was performed as previously described [19]. We used the following primary antibodies: anti-CD81 (Abcam, Cat# ab79559), anti-TSG101 (Abcam, Cat# ab125011), anti-E-cadherin (Cell Signaling Technology, Danvers, MA, USA, Cat# 14472S), anti-Vimentin (Abcam, Cat# ab92547), anti-N-cadherin (Cell Signaling Technology, Cat# 13116S), anti-MMP-2 (Cell Signaling Technology, Cat# 40994S), anti-MMP-9 (Cell Signaling Technology, Cat# 13667T), anti-ZEB1 (Cell Signaling Technology, Cat# 70512S), anti-Snail (Cell Signaling Technology, Cat# 3879S), anti-Slug (Cell Signaling Technology, Cat# 9585S) anti-Twist1 (Cell Signaling Technology, Cat# 46702S), anti-EGFR (Cell Signaling Technology, Cat# 4267S), anti-ERK antibody (Cell Signaling Technology, Cat# 4696S) and anti-p-ERK antibody (Cell Signaling Technology, Cat# 4370T), anti-androgen receptor (Abcam, Cat# ab74272), anti-β-actin (Cell Signaling Technology, Cat# 3700S) and anti-GAPDH (Cell Signaling Technology, Cat# 5174S).

### Gelatin zymography

Gelatin zymography was performed as previously described [26]; however, we made some modifications. Briefly, LNcaP and DU145 cells were incubated with CAFs-derived exosomes or/and transfected with miRNA mimics, then cultured in serum-free medium. Cell culture supernatants were gathered after 24 h and centrifuged at 2000rpm for 10 min. Protein concentration was measured by the BCA method. Samples were mixed with a 2× nonreducing loading buffer and 8% sodium dodecyl sulfate (SDS) containing 1mg/ml gelatin was used to electrophorese. Then we performed the gelatin zymography by using MMP Zymography assay kit (Applygen, P1700, Applygen Technologies Inc, Beijing, China).

### Transwell migration and invasion assay

Transwell chambers with 8 μM pore size (Corning, Costar 3464, Corning, NY, USA) were performed to evaluate the migration and invasion ability of LNCaP and DU145 in the presence or absence of CAFs-derived exosomes. LNCaP and DU145 were pretreated with 10% CSFBS DMEM medium for 24 h in advance, and then incubated with CAFs-derived exosomes at a concentration of 25 μg/mL in 10% CSFBS DMEM medium for 48 h as previously described [27]. For the migration assay, 8×10^4^ LNCaP cells or 3×10^4^ DU145 cells were mixed in 100 μl of serum-free medium and seeded onto the upper chambers of the Transwell, DMEM medium with 10% CSFBS was placed in the lower chambers. After 48 h, LNCaP and DU145 cells that migrated through the membrane were stained with crystal violet. To assess invasion ability of LNCaP and DU145 cells, Matrigel (BD Biosciences, San Jose, CA, USA) in serum-free medium was added on top of the Transwell membrane and allowed to dry for 1 h at 37 °C. Then 8×10^4^ LNCaP cells or 3×10^4^ DU145 cells were seeded, after 72 h and 24 h, invading LNCaP and DU145 cells at the Transwell membrane were labelled, respectively.

### RNA extraction and quantitative real-time PCR (qRT-PCR)

Total RNA of isolated exosomes, cells and tissues were extracted using Trizol (Takara, Japan) reagent following the procedure. Reverse transcription was performed using Prime Script RT reagent Kit (Takara). The real-time PCR was conducted with TB Green TM Premix Ex Taq TM (Takara) on Quant Studio 6 Flex (Applied Biosystems). All procedures above followed standard instructions. We used Bulge-loop™ miRNA qRT-PCR Primer Sets (one RT primer and a pair of qPCR primers for each set) to determine miRNA quantification. Primers and oligos were supplied by RiboBio (Guangzhou, China) and Sangon Biotech (Shanghai, China) and sequences of some primers are listed in Additional file 8: Table S1. The relative expression of mRNA or miRNA was determined using the 2^-ΔΔCt^ method. All results are representative of three independent experiments. And we used GAPDH as the reference of mRNA and U6 as the reference of miRNA.

### miRNA sequencing

Exosomes were isolated from three sets of primary CAFs, total RNA was isolated from exosomes. Library construction, miRNA sequencing, and bioinformatics data analysis were performed by CloudSeq Biotech (Shanghai, China). In brief, the amount and purity of RNA were analysed by a NanoDrop ND-100 (Thermo Fisher Scientific). Only small RNAs of length 20-22 nt were selected to prepare libraries and PCR amplification. Then the products were sequenced via Illumina HiSeq sequencer (Illumina, USA). Differentially expressed miRNAs were analysed by a twofold change and a significant *p*-value (0.05).

### Xenograft models and bioluminescence imaging in vivo

All animal procedures were performed in accordance with the guidelines of laboratory animals and approved by the Institutional Animal Care and Use Committees of Shanghai General Hospital. 4 to 6-week-old male BALB/c nude mice (Beijing Vital River, Beijing, China) were maintained under specific pathogen-free conditions in the animal centre of Shanghai General Hospital. To evaluate the effect of DHT/ETOH-treated CAFs-derived exosomes on tumour metastasis, the nude mice were divided into 3 groups with 5 mice in each group at random and surgical castration was performed as previously described [28]. A week later, we performed injections of DU145 luciferase (DU145-Luc) cells. Before tumour cell injection, DU145-Luc cells were pre-incubated with DHT-treated CAFs-derived exosomes (25 μg/mL), ETOH-treated CAFs-derived exosomes (25 μg/mL), or PBS twice a day for 4 days. Then, we injected the indicated cell lines (10^6^ cells in 100ul PBS per mouse) through the tail vein. Thereafter, according to a recent study [29], mice were treated with CAFs-derived exosomes (150μg in 100ul PBS per mouse) or PBS via tail vein injections every other day for 2 weeks. 4 or 8 weeks after the injection of tumour cells, tumour metastasis was observed by a bioluminescence-based in vivo imaging system (IVIS, Caliper Life Science, MA, USA). The mice were anesthetized with 1.5% isoflurane/air and intraperitoneally injected with D-luciferin (200μl at 15mg/ml in PBS) before imaging. Afterward, the mice were sacrificed 8 weeks after tumour injection, we removed the lungs and captured the images. The tumour foci in lungs were collected and quantified, and some metastatic tumours were fixed in 4% paraformaldehyde and paraffin-embedded for haematoxylin and eosin (H&E) and immunohistochemistry.

### Detection of transfer of miR-146a-5p via exosomes in vitro

To examine whether CAFs-derived exosomal miR-146a-5p could be transferred to PCa cells, Cy3-labeled miR-146a-5p or negative control was transfected to CAFs, then we cultured CAFs in complete medium containing exosome-free FBS for 48 h, and the conditioned medium was added to DU145 for 24 h. Then the DU145 cells were fixed for immunofluorescence. Moreover, the cytoskeleton of DU145 was stained with Vimentin and the nuclei were stained with 1 × hoechest 33,342. Finally, fluorescent microscopy was used to detect the green signals (Vimentin) and red fluorescent signals (Cy3-labeled miR-146a-5p) in DU145.

To determine uptake of exosomes, CAFs were transfected with Cy3-labelled miR-146a-5p or negative control for 24 h, followed by washing with PBS and cultured in complete medium containing exosome-free FBS for 48 h. CAFs-derived exosomes were isolated as described above. The exosomes were suspended in freshly medium and added to DU145. After 24 h, DU145 were fixed with 4% paraformaldehyde for further observation.

### Dual luciferase reporter assay

Luciferase reporter constructs encoding NC 3’UTR, the wild-type EGFR 3’UTR region, or mutant EGFR 3’UTR region were synthesized by Genomeditech. In brief, 293 T cells were seeded into a 24-well plate, then were co-transfected with luciferase reporter, miR-NC mimics/miR-146a-5p mimics, and Renilla luciferase vector (pRL-TK; Genomeditech) by using HG transgene reagent (Genomeditech). After 48 h, luciferase activities were detected using a Dual-Luciferase Reporter Assay kit (Genomeditech) following the manufacturer’s protocol.

### Immunohistochemistry (IHC)

IHC was performed as described previously [30]. Tissues were embedded in paraffin and IHC was performed using anti-E-cadherin antibody (Cell Signaling Technology, Cat# 14472S), anti-Vimentin antibody (Abcam, Cat# ab92547), anti-EGFR antibody (Cell Signaling Technology, Cat# 4267S), anti-ERK antibody (Cell Signaling Technology, Cat# 4696S) and anti-p-ERK antibody (Cell Signaling Technology, Cat# 4370T).

### Proliferation assay

Cell viability was determined by Cell Counting Kit-8 (CCK-8, Dojindo, Kumamoto, Japan). Cell proliferation was measured by 5-ethynyl-29-deoxyuridine (EdU) assay (RiboBio, Guangzhou, China). For the CCK-8 assay, cells were pretreated with exosomes derived from ETOH-treated CAFs or DHT-treated CAFs for 48 h, then seeded in 96-well plate in triplicate at a concentration of 1000 cells each well, and cells were treated with indicated exosomes or PBS every other day. At different time points, 10 μl CCK-8 solution was added to each well and incubated at 37 °C for 2 h. The absorbance was detected at 450 nm using a microplate reader (Bio-Rad Laboratories). EdU assays were performed to determine the proliferation of LNcaP and DU145 cells after incubation with different exosomes for 48 h according to the protocol.

### Statistical analysis

The data were presented as mean ± standard deviation (SD). Statistical analysis was performed using GraphPad Prism 7 software. A two-tailed *t*-test was used to assess the differences. *p* < 0.05 indicated a significant difference.

## Results

### Characteristics of CAFs isolated from PCa patients and isolation of exosomes

**Fig. 1:**
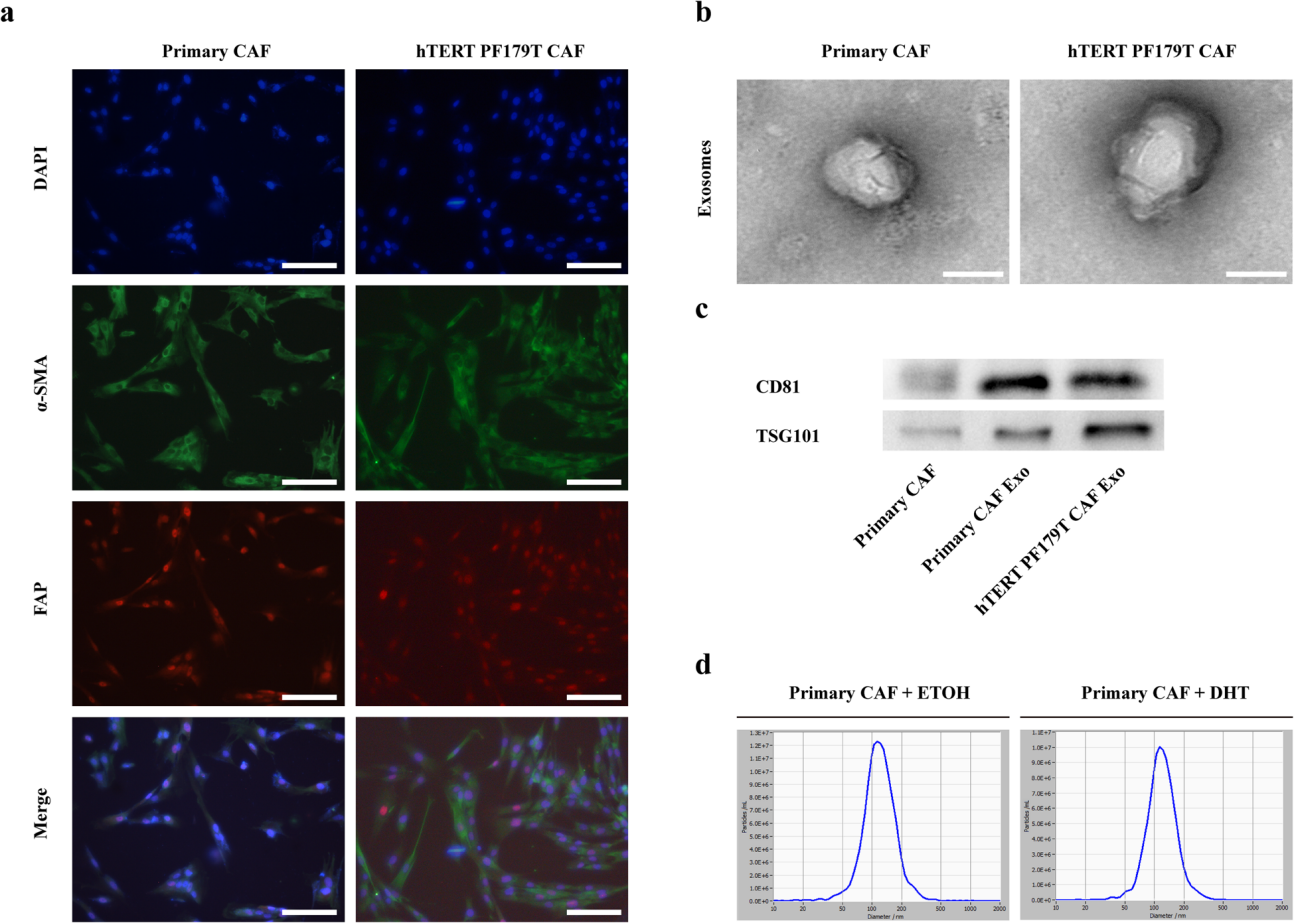
Characteristics of CAFs derived from PCa patients and isolation of exosomes. **a** Immunofluorescence staining for α-SMA and FAP of primary CAFs and hTERT PF179T CAF (scale bars = 25 μm). **b** Exosomes derived from CAFs were analysed by transmission electron microscopy (TEM) (scale bars = 25 nm). **c** The expression of exosomal positive markers CD81 and TSG101 in CAFs and CAFs-derived exosomes were evaluated by western blot. **d** Nanoparticle Tracking Analysis showing the size and concentration of exosomes from ETOH-treated CAFs or DHT-treated CAFs. Data are shown as mean ± SD representing triplicate measurements. (Student’s t-test, *P < .05, **P < .01)

We isolated and cultured three sets of primary CAFs from PCa samples. Next, we characterized the phenotypes of primary CAFs and hTERT PF179T CAF cell line. We performed immunofluorescence to detect the expression of CAF markers such as α-SMA, FAP, and Vimentin (Fig. 1a and Additional file 2: Figure S2a). we used western blotting assay to detect the androgen receptor (AR) expression in CAFs and found that AR was expressed in all CAFs but at a lower level than LNCaP cells (Additional file 2: Figure S2b).

CAF-secreted exosomes could contribute to the progression and metastasis by transferring different types of substances in various tumours [31]. Previous study showed that inhibiting AR signalling in CAFs might promote PCa cell migration by enhanced secretion of CCL2 and CXCL8 [11], we would like to determine whether exosomes derived from CAF exposed to androgen deprivation might promote migration or invasion of PCa cells. Exosomes were isolated from the conditioned medium of CAFs. Firstly, the exosomes were resuspended with PBS and characterized by transmission electron microscopy (TEM). As shown in Fig. 1b, particles isolated from the conditioned medium of CAFs ranged from 30 to 150 nm. Secondly, western blotting analysis of purified exosomes revealed the expression of the exosomal positive markers CD81 and TSG101 (Fig. 1c). Lastly, the effect of DHT treatment on exosome release in CAFs were assessed. We used Nanoparticle Tracking Analysis to confirm the size and concentration of released exosomes. Upon DHT treatment, CAFs did not display increased levels of exosome secretion (Fig. 1d). Based on the results above, we verified that the particles isolated from the supernatants of CAFs were exosomes and DHT did not influence the number of exosomes released from CAFs.

### Exosomes from CAFs after ADT increase migration and invasion of PCa cells via activating EMT both in vitro and in vivo

**Fig. 2:**
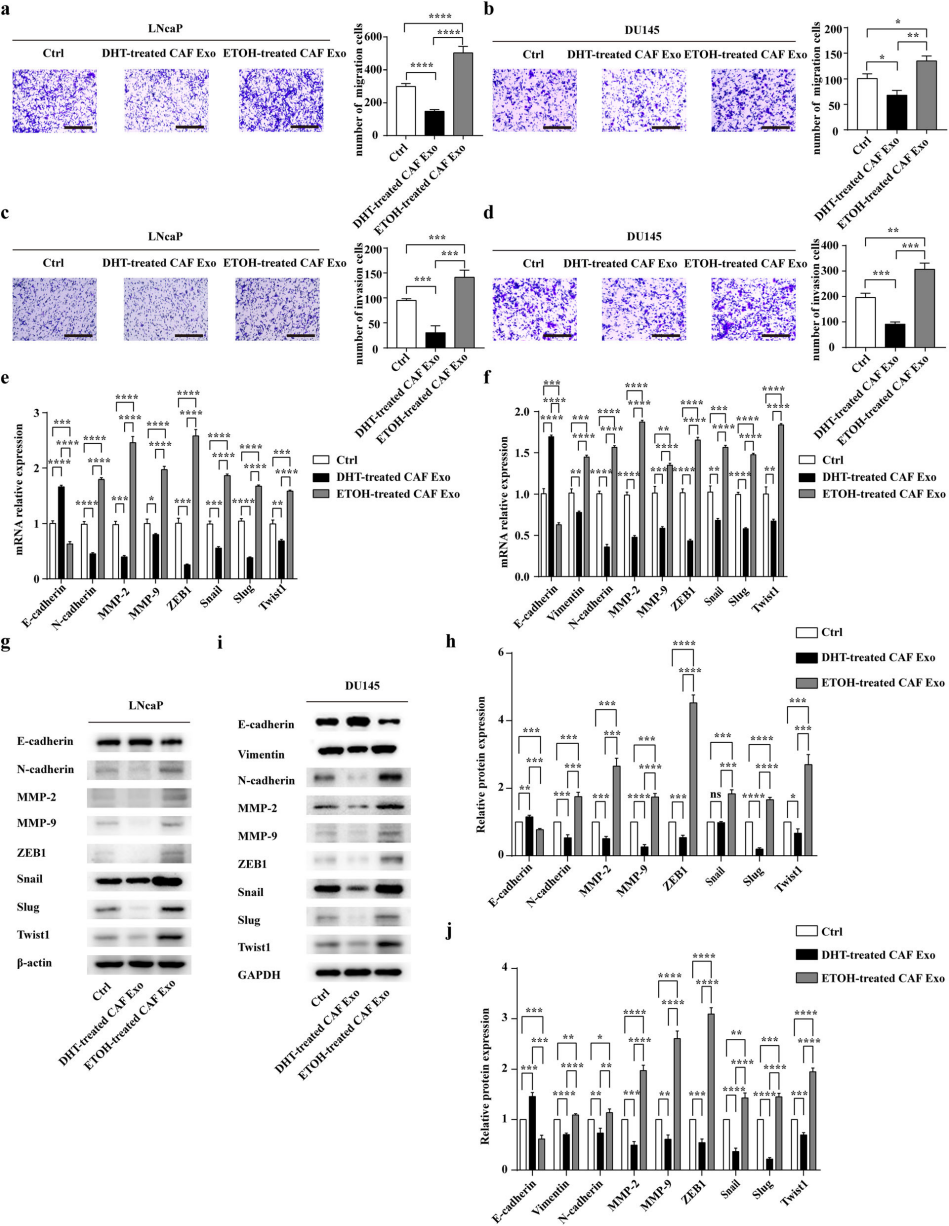
Exosomes from CAFs after ADT promote EMT, migration and invasion of PCa cells in vitro. **a** and **b** Relative cell migration ability was evaluated using a Transwell assay (scale bars = 25 μm). Before that, LNCaP and DU145 were co-cultured with different exosomes (25 μg/mL) for 2 days under castration condition. **c** and **d** The effect of different exosomes on the invasion of both LNCaP and DU145 cells was assessed by a Transwell assay (scale bars = 25 μm). **e** and **f** LNCaP and DU145 cells were incubated with exosomes derived from ETOH-treated CAFs or DHT-treated CAFs for 48 h, and the RNA levels of EMT markers and MMPs were analysed by qRT-PCR. **g** and **h** LNCaP cells were incubated with exosomes derived from ETOH-treated CAFs or DHT-treated CAFs for 48 h, and Western blotting and densitometric quantification analysed the protein levels of EMT markers and MMPs. i and j: DU145 cells were incubated with exosomes derived from ETOH-treated CAFs or DHT-treated CAFs for 48 h, and western blotting and densitometric quantification analysed the protein levels of EMT markers and MMPs. Data are shown as mean ± SD representing triplicate measurements. (Student’s t-test, *P < .05, **P < .01, ***P < .001, ****P < .0001)

We next evaluated the effect of CAFs-derived exosomes on PCa cell migration and invasion while inhibiting AR signalling in CAFs. Exosomes were collected as described and added to recipient cells in freshly 10% CSFBS DMEM medium for 48 h. Compared with DHT-treated CAFs-derived exosomes (DHT-treated CAF Exo), ETOH-treated CAFs-derived exosomes (ETOH-treated CAF Exo) significantly enhanced the migration and invasion ability of LNCaP and DU145 (Figs. 2a-d). It is well known that EMT-markers and matrix metalloproteinases (MMPs) are involved in metastasis of cancer cells [32]. Therefore, we detected the RNA and protein expressions of EMT markers (E-cadherin, Vimentin, N-cadherin, ZEB1, Snail, Slug, Twist1) and MMPs in PCa cells (Fig. 2e-j). Due to the low expression of Vimentin in LNcaP cells, we only detected the RNA and protein expression of Vimentin in DU145. The results showed that PCa cells in the ETOH-treated CAF Exo group expressed lower level of E-cadherin than in the DHT-treated CAF Exo group (Fig. 2e-j). And we also found DHT-treated CAFs-derived exosomes decreased the expression of other proteins including Vimentin, N-cadherin, MMP-2, MMP-9, ZEB1, Snail, Slug, Twist1 in PCa cells than the control group; however, exosomes isolated from ETOH-treated CAFs increased the expression of those markers in PCa cells than that in the DHT-treated CAF Exo group (Fig. 2e-j). In addition, zymograms showed that ETOH-treated CAFs-derived exosomes enhanced the activities of MMP-2 and MMP-9 in PCa cells than that in the DHT-treated CAF Exo group (Additional file 3: Figure S3a-f).

**Fig. 3:**
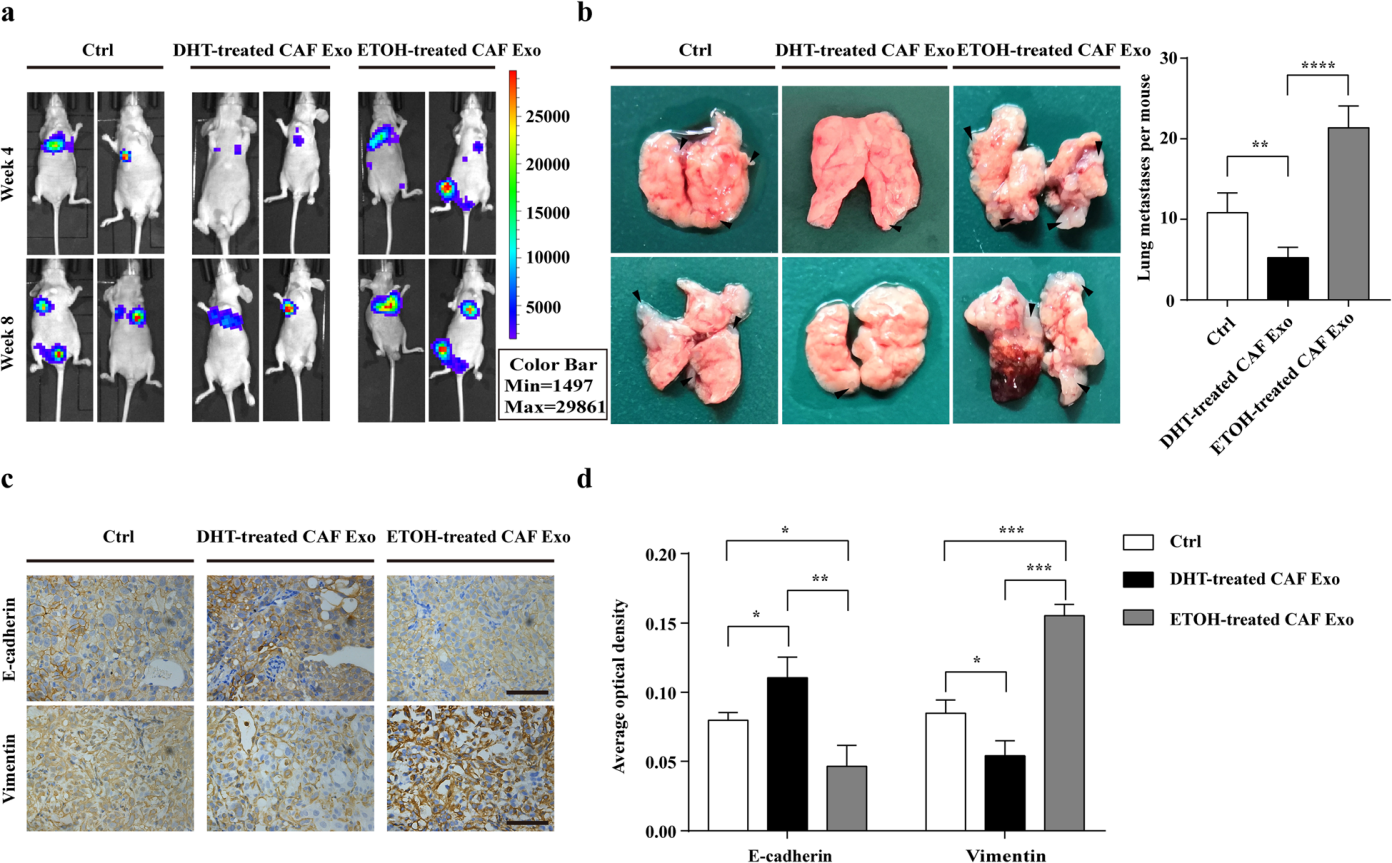
Exosomes from CAFs after ADT promote EMT, migration and invasion of PCa cells in vivo. **a** Representative bioluminescence images of BALB/c nude mice that were injected with DU145 luciferase cells through tail vein and treated with either CAFs-derived exosomes or PBS via tail vein injections every other day for 2 weeks. Prior to tail vein injection, DU145 luciferase cells were pre-incubated with either CAFs-derived exosomes or PBS twice a day for 4 days. Images were taken in week 4 and week 8 (*n* = 5). **b** Tumour foci in lungs of mice after injecting the indicated DU145 cells and exosomes/PBS through tail vein. The right graph shows the number of lung metastases per mouse. **c** Immunohistochemical staining of E-cadherin and Vimentin in the lung metastases of ETOH/DHT-treated CAF Exo group and the control group (scale bars = 12.5 μm). **d** Average optical density of immunostaining of E-cadherin and Vimentin in the lung metastases of ETOH/DHT-treated CAF Exo group and the control group. Data are shown as mean ± SD representing triplicate measurements. (Student’s t-test, * *P* < .05, **P < .01, ***P < .001, ****P < .0001)

To confirm the effects of exosomes derived from CAFs exposed to ADT on PCa metastasis in vivo, androgen ablation was induced by surgical castration in mice. Prior to tail vein injection, DU145 luciferase cells were pre-incubated with exosomes derived from DHT/ETOH-treated CAFs or PBS twice a day for 4 days. Then we injected the indicated cell lines (10^6^ cells in 100ul PBS per mouse) through the tail vein. After injection of tumour cells, mice were treated with CAFs-derived exosomes or PBS via tail vein injections every other day for 2 weeks. Then, bioluminescence imaging was employed to monitor tumour metastasis. Consistent with the experiments in vitro, luciferase signals and the number of lung metastases were higher in the ETOH-treated CAF Exo group than DHT-treated CAF Exo group and the control group (Fig. 3a, b). Significantly, immunohistochemical analysis demonstrated that the expression level of E-cadherin was lower, while the abundance of Vimentin was higher in ETOH-treated CAFs Exo group (Fig. 3c, d).

In addition, we assessed the effect of CAFs-derived exosomes on PCa cell proliferation under castration condition. However, we found that ETOH/DHT-treated CAFs-derived exosomes did not affect the proliferation of PCa cells under castration condition (Additional file 4: Figure S4a-d).

In conclusion, after androgen deprivation therapy, exosomes from CAFs might promote the migration and invasion of PCa cells via activating EMT both in vitro and in vivo.

### The level of miR-146a-5p significantly decreased in the exosomes isolated from CAFs after ADT

**Fig. 4:**
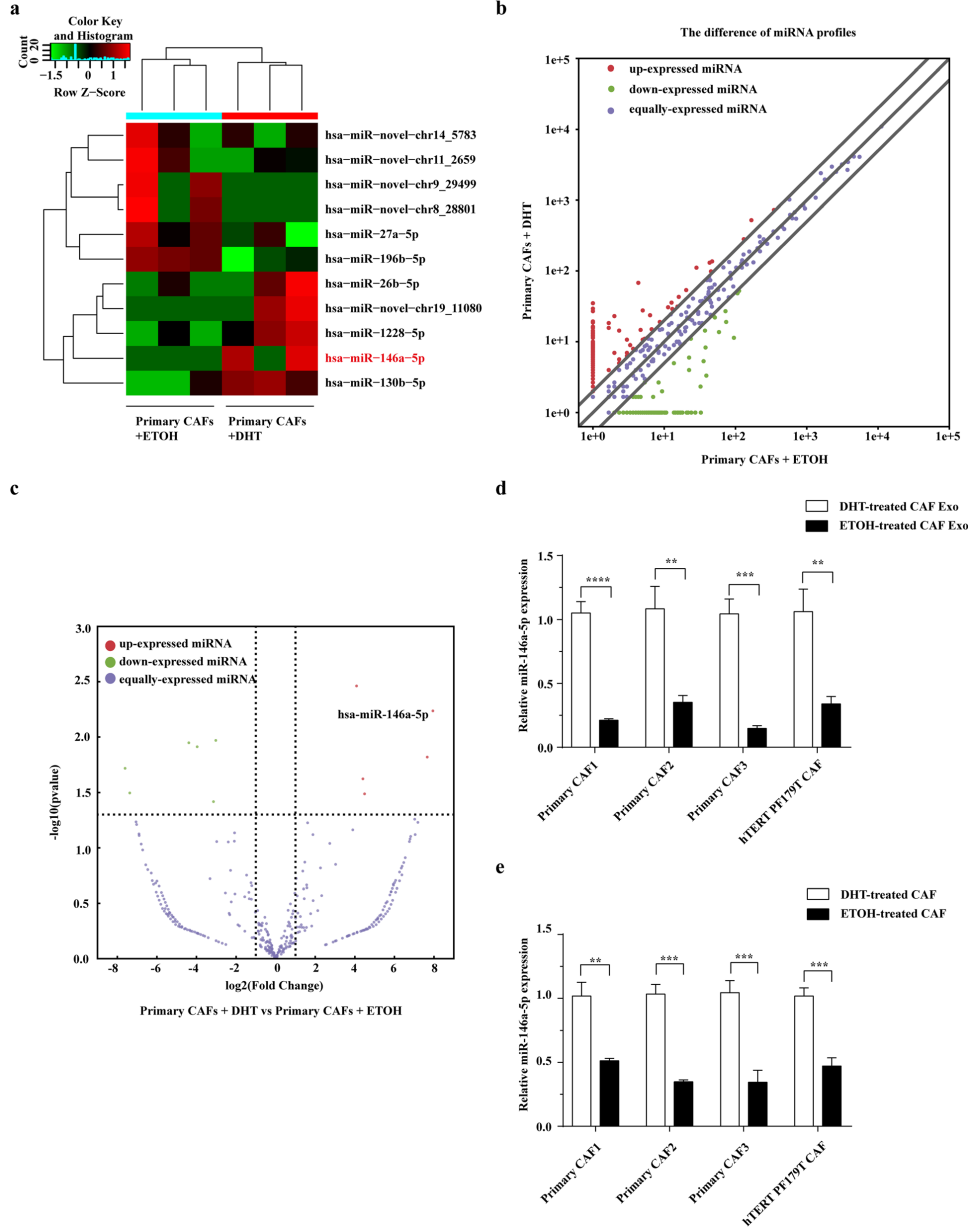
The level of miR-146a-5p significantly decreased in the exosomes isolated from CAFs after ADT. **a** Heatmap of the significantly differential miRNAs between ETOH-treated and matched DHT-treated CAFs-derived exosomes. Red, green, and black indicated miRNAs with higher, lower, and equal expression, respectively. **b** The scatter plot represents the differential miRNA expression profiles between ETOH-treated and DHT-treated CAFs-derived exosomes. Red, green, and purple indicated miRNAs with higher, lower, and equal expression, respectively. **c** Volcano plot presents differential expression of miRNAs between ETOH-treated and matched DHT-treated CAFs-derived exosomes. Red, green, and purple indicated miRNAs with higher, lower, and equal expression, respectively. **d** Real-time PCR analysis of miR-146a-5p expression in exosomes from DHT-treated CAFs and matched ETOH-treated CAFs (*n* = 4). **e** Real-time PCR analysis showing the expression of miR-146a-5p in DHT-treated CAFs and matched ETOH-treated CAFs (*n* = 4). Data are shown as mean ± SD representing triplicate measurements. (Student’s t-test, **P < .01, ***P < .001, ****P < .0001)

MiRNAs are principal and important cargos in exosomes. To figure out the molecular mechanism of ETOH-treated CAFs-derived exosomes promoting migration and invasion of PCa cells, we performed miRNA sequencing to analyse the miRNA profiles of exosomes derived from DHT-treated CAFs and matched ETOH-treated CAFs. As shown in Figs. 4a-c, 6 miRNAs were significantly upregulated, and 5 miRNAs were significantly downregulated in the ETOH-treated CAFs-derived exosomes. Significantly, we observed that the expression of miR-146a-5p had a marked decrease in ETOH-treated CAFs-derived exosomes. Therefore, we performed real-time PCR to confirm the sequence result. The result showed that the level of miR-146a-5p was significantly lower in exosomes derived from ETOH-treated CAFs than that of exosomes derived from DHT-treated CAFs (Fig. 4d). Furthermore, the level of miR-146a-5p was also downregulated in ETOH-treated CAFs compared with matched DHT-treated CAFs (Fig. 4e). Therefore, among these miRNAs, we chose miR-146a-5p for further study.

### Exosomes transfer miR-146a-5p from CAFs to PCa cells

**Fig. 5:**
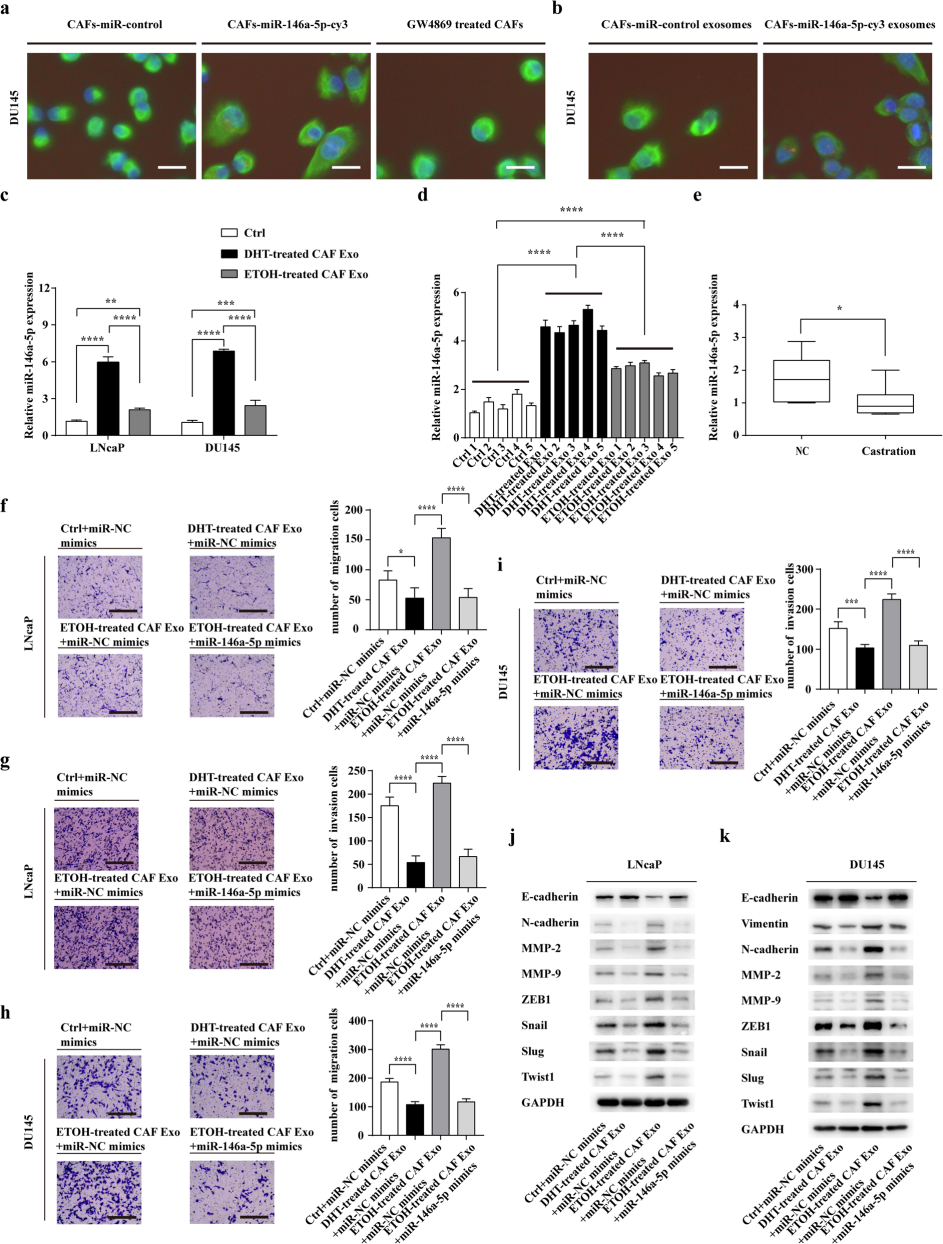
Exosomes transfer miR-146a-5p from CAFs to PCa cells. **a** CAFs transiently transfected with Cy3-tagged miR-146a-5p or with the miR-control, and GW4869-treated CAFs transfected with Cy3-tagged miR-146a-5p were co-cultured with DU145 for 24 h. Fluorescence microscopy was performed to observe the green signals (Vimentin) and red fluorescent signals (Cy3) in DU145 (scale bars = 6.25 μm). **b** Exosomes were isolated from the supernatants of CAFs transfected with cy3-tagged miR-146a-5p (CAFs-miR-146a-5p-cy3 exo) or with miR-control (CAFs-miR-control exo) and exosomes (25 μg/mL) were added to DU145 for 24 h. Fluorescence microscopy was performed to detect the green signals (Vimentin) and red fluorescent signals (Cy3) in DU145 (scale bars = 6.25 μm). **c** The expression of miR-146a-5p in LNcaP and DU145 cells were measured using Real-time PCR at 24 h after treating with exosomes (25 μg/mL) derived from DHT/ETOH-treated CAFs. **d** The expression of miR-146a-5p in lung metastasis of the nude mice among three groups by Real-time PCR. **e** The level of miR-146a-5p in androgen deprived prostate tissues (Castration) was compared with that of the control PCa tissues (NC) (*n* = 8) by Real-time PCR. **f** and **g** LNcaP cells were co-cultured with CAFs-derived exosomes and transfected with miR-NC or miR-146a-5p mimics for 48 h, and the migration and invasion of LNcaP cells was assessed by a Transwell assay (scale bars = 25 μm). **h** and **i** DU145 cells were co-cultured with CAFs-derived exosomes and transfected with miR-NC or miR-146a-5p mimics for 48 h, and the migration and invasion of DU145 cells was assessed by a Transwell assay (scale bars = 25 μm). **j** and **k** LNcaP and DU145 cells were co-cultured with CAFs-derived exosomes and transfected with miR-NC or miR-146a-5p mimics for 48 h, and western blotting analysed the protein levels of EMT markers and MMPs. Data are shown as mean ± SD representing triplicate measurements. (Student’s t-test, * *P* < .05, **P < .01, ***P < .001, ****P < .0001)

To determine if miR-146a-5p could be transferred via exosomes from CAFs to PCa cells, CAFs were transiently transfected with Cy3-labeled miR-146a-5p, then co-cultured with DU145 for 24 h. We used fluorescence microscopy to detect the red signals (Cy3-labeled miR-146a-5p) and green signals (Vimentin) in DU145. Cy3-tagged miR-146a-5p was observed in DU145 and the red signals were abolished when treating CAFs with the exosome inhibitor, GW4869 (Fig. 5a and Additional file 5: Figure S5).

We then isolated exosomes from CAFs transfected with Cy3-labeled miR-146a-5p. The purified exosomes were added to DU145 and incubated for 24 h. After that, we detected the red fluorescent signals in DU145 with fluorescence microscopy (Fig. 5b). In conclusion, these results suggested that miR-146a-5p could be transferred from CAFs to PCa cells via exosomes.

Significantly, RT-PCR analysis showed that miR-146a-5p expression was decreased in PCa cells after treating with exosomes from ETOH-treated CAFs than that of cells treated with DHT-treated CAFs-derived exosomes (Fig. 5c). In addition, we analysed the expression of miR-146a-5p in lung metastasis of the nude mice among three groups. It showed that the expression of miR-146a-5p in ETOH-treated CAF Exo group was much lower than that in DHT-treated CAF Exo group (Fig. 5d). Furthermore, RT-PCR analysis confirmed that the expression of miR-146a-5p was much lower in the tumours from patients who received ADT than that of tumours from patients who underwent operations without ADT therapy (Fig. 5e). Taken together, CAFs may decrease the expression of miR-146a-5p in PCa cells through downregulating the transfer of exosomal miR-146a-5p after ADT.

To determine whether ETOH-treated CAFs-derived exosomes promote the migration and invasion ability of PCa cells via downregulating the transfer of miR-146a-5p. PCa cells were co-cultured with ETOH-treated CAFs-derived exosomes, at the same time, PCa cells were transfected with miR-NC or miR-146a-5p mimics. The results revealed that compared with the miR-NC group, miR-146a-5p overexpression reversed the effect of ETOH-treated CAFs-derived exosomes on migration and invasion of LNcaP and DU145 (Fig. 5f-i). Western blot assay verified the protein expressions of EMT markers and MMPs in LNcaP and DU145 (Fig. 5j, k), and gelatin zymography showed that miR-146a-5p overexpression reversed the effect of ETOH-treated CAFs-derived exosomes on the activities of MMPs in LNcaP and DU145 (Additional file 6: Figure S6a-f).

### MiR-146a-5p impairs cell migration and invasion by inhibiting EMT in PCa cells

**Fig. 6:**
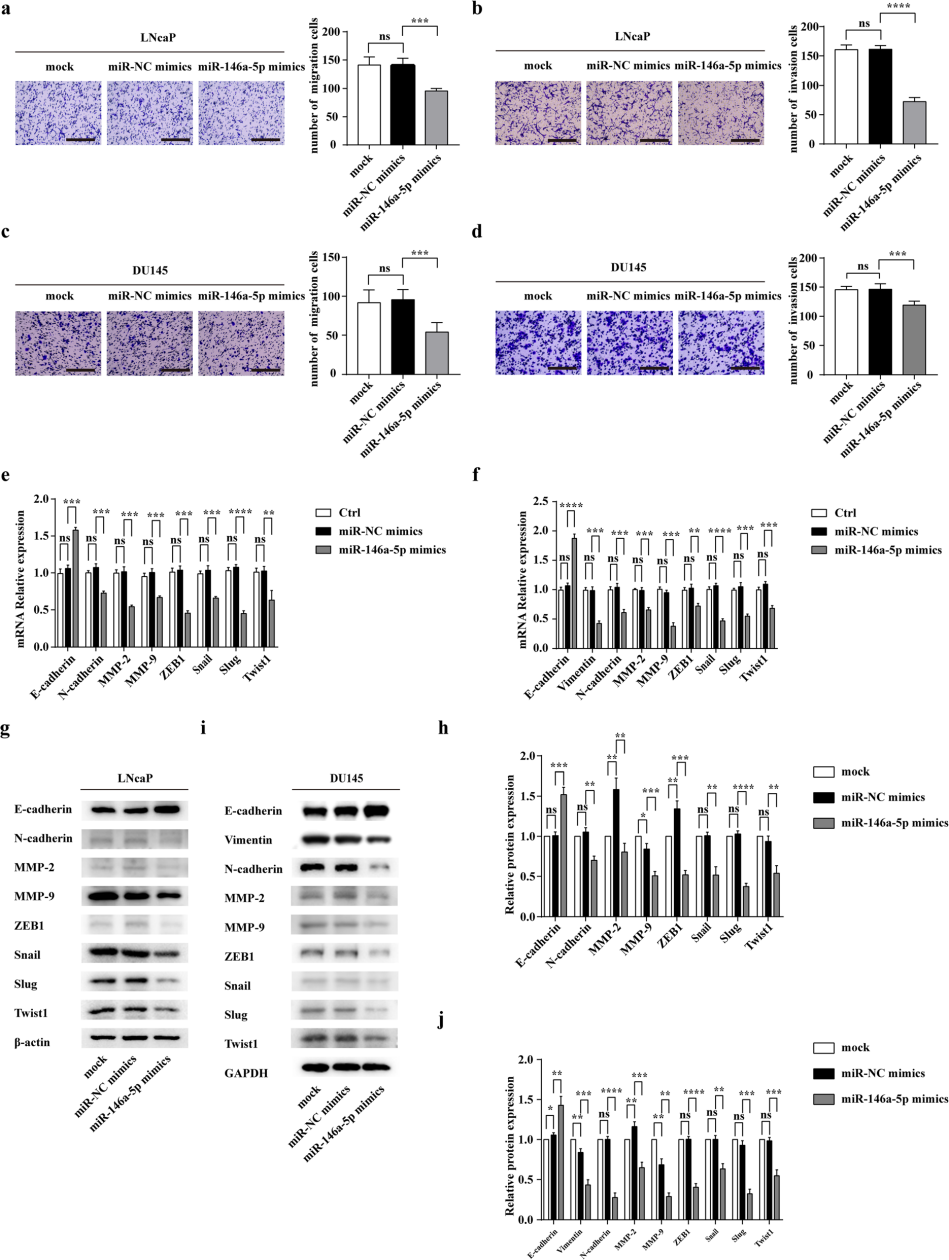
MiR-146a-5p impairs cell migration and invasion by inhibiting EMT in PCa cells in vitro. **a** and **b** The effect of miR-146a-5p on the migration and invasion of LNcaP cells was assessed by a Transwell assay (scale bars = 25 μm). **c** and **d** The effect of miR-146a-5p on the migration and invasion of DU145 cells was assessed by a Transwell assay (scale bars = 25 μm). **e** and **f** The effect of miR-146a-5p on the RNA levels of EMT markers and MMPs in LNcaP and DU145 cells. **g** and **h** The effect of miR-146a-5p on the protein levels of EMT markers and MMPs in LNcaP were determined by western blot and densitometric quantification. **i** and **j** The effect of miR-146a-5p on the protein levels of EMT markers and MMPs in DU145 were determined by western blot and densitometric quantification. Data are shown as mean ± SD representing triplicate measurements. (Student’s t-test, ***P < .001, ****P < .0001)

To determine whether miR-146a-5p influences the migration and invasion of PCa cells, we transfected cells with miR-146a-5p mimics and conducted a Transwell assay. Interestingly, we found that miR-146a-5p overexpression decreased the migration and invasion of both LNcaP and DU145 cells (Figs. 6a-d). In addition, western blotting and PCR assay showed that relative expression of E-cadherin was increased in both LNcaP and DU145 cells after transfection of miR-146a-5p mimics (Fig. 6e-j). And we found that miR-146a-5p suppressed not only the expressions of other EMT markers (Vimentin, N-cadherin, ZEB1, Snail, Slug, Twist1), but also the expressions and activities of MMPs in LNcaP and DU145 (Fig. 6e-j and Additional file 7: Figure S7a-f). In brief, miR-146a-5p impairs the migration and invasion by inhibiting EMT in PCa cells.

### EGFR is a direct target of exosomal miR-146a-5p in PCa cells

**Fig. 7:**
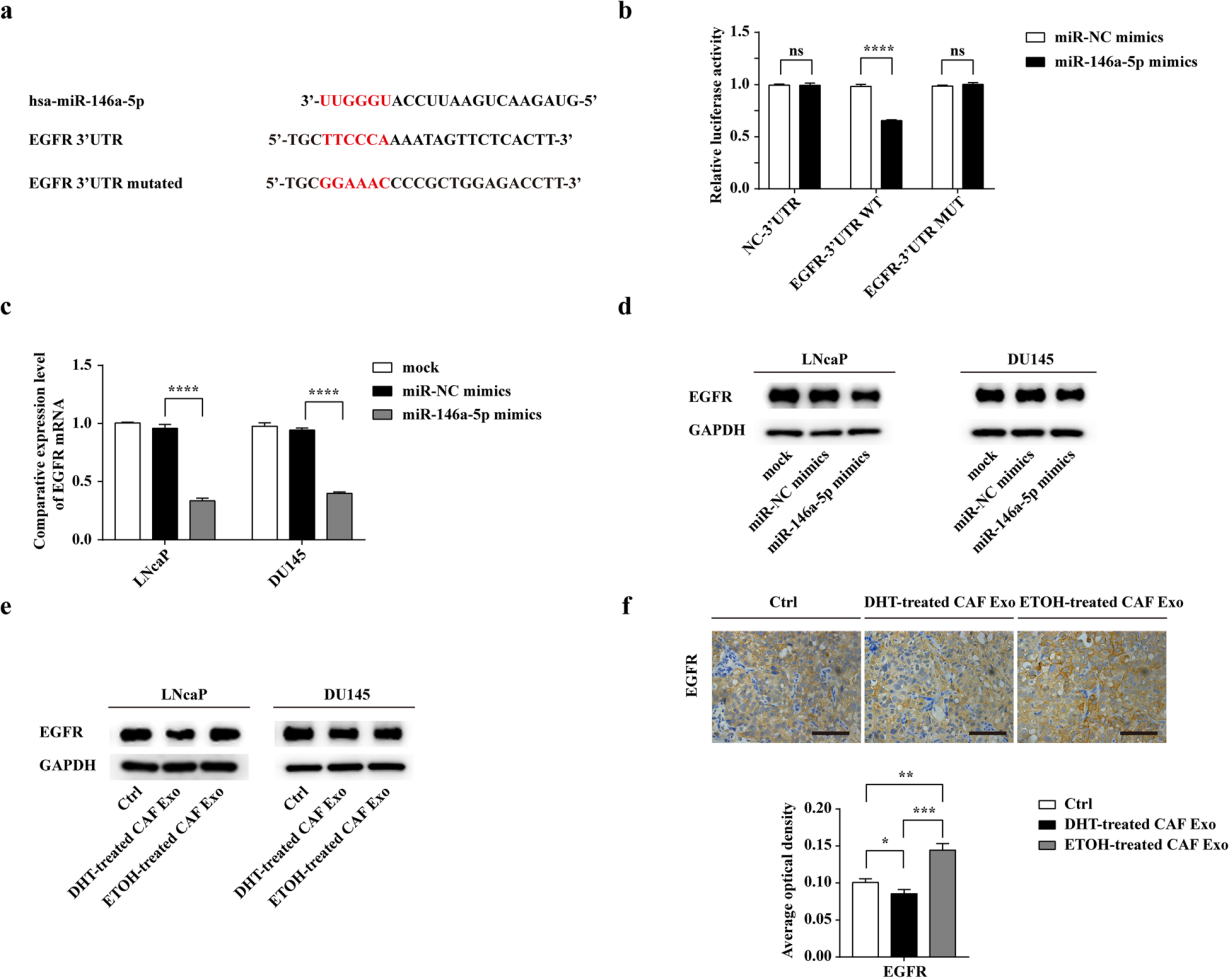
EGFR is a direct target of exosomal miR-146a-5p in PCa cells. **a** Predicted miR-146a-5p target sequences in the 3′ UTRs of EGFR and constructions of wild-type and mutant EGFR 3′-UTR luciferase reporter. **b** The relative luciferase activities were measured after co-transfection of miR-NC mimics or miR-146a-5p mimics and a luciferase vector encoding NC 3’UTR, the wild-type, or mutant EGFR 3’UTR region in 293 T cells. **c** Quantitation of EGFR mRNA expression in LNcaP and DU145 transfected with miR-NC mimics and miR-146a-5p mimics by real-time PCR. **d** EGFR expression in PCa cells was detected by western blot analysis at 48 h after transfection of miR-NC mimics or miR-146a-5p mimics. **e** Exosomes from ETOH/ DHT-treated CAFs were isolated and added to LNcaP and DU145 (25 μg/ml) for 48 h, Western blot showed the protein levels of EGFR in PCa cells at different conditions. **f** Immunohistochemical staining and average optical density of EGFR in the lung metastases of ETOH/DHT-treated CAF Exo group and the control group (scale bars = 12.5 μm). Data are shown as mean ± SD representing triplicate measurements. (Student’s t-test, * *P* < .05, **P < .01, ***P < .001, ****P < .0001)

To delineate the potential targets of miR-146a-5p in prostate cancer, we used publicly available bioinformatics tools (miRWalk, miRTarBase) to predict 89 target genes. Among these genes, EGFR was reported to promote prostate cancer bone metastasis [33]. Using miRTarBase, there are three predicted binding sites of miR-146a-5p in EGFR 3’UTR region and we designed a luciferase vector containing a wild-type or mutant EGFR 3’UTR region (Fig. 7a). To verify whether miR-146a-5p could target the 3’UTR of EGFR directly, a dual luciferase reporter assay was conducted. The result showed that the luciferase activities of EGFR WT 3’UTR could be significantly reduced after co-transfection with miR-146a-5p mimics compared with co-transfection with miR-NC mimics. In contrast, no significant direct interaction was observed between miR-146a-5p and the vector containing EGFR MUT 3’UTR (Fig. 7b).

In both LNcaP and DU145, overexpression of miR-146a-5p markedly inhibited the EGFR mRNA level (Fig. 7c). Additionally, western blot analysis showed that transfection of miR-146a-5p repressed the protein expression of EGFR in both LNcaP and DU145 cells (Fig. 7d). As expected, after incubation with CAFs-derived exosomes, the protein level of EGFR in both cell lines were increased in ETOH-treated CAF Exo group than that in the DHT-treated CAF Exo group (Fig. 7e). Moreover, IHC analysis confirmed that EGFR staining was significantly stronger in lung metastasis of mice treated with ETOH-treated CAFs-derived exosomes than that of mice treated with DHT-treated CAFs-derived exosomes (Fig. 7f). Together, EGFR is a direct target of exosomal miR-146a-5p in PCa cells.

### Exosomal miR-146a-5p exhibits its functions by inhibiting EGFR/ERK pathway in PCa cells

**Fig. 8:**
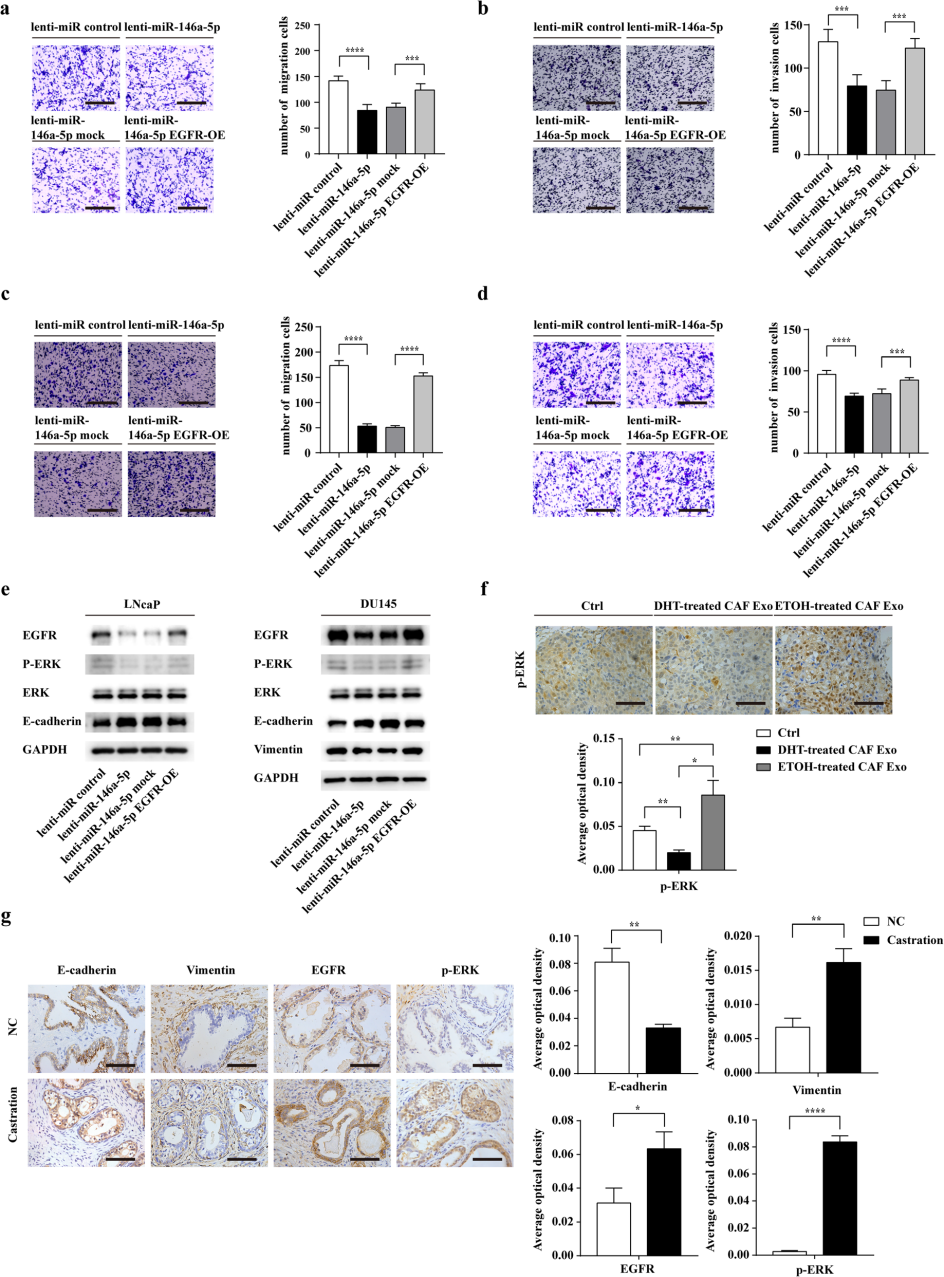
Exosomal miR-146a-5p exhibits its functions by inhibiting EGFR/ERK pathway in PCa cells. **a** and **b** Migration and invasion of LNcaP cells transfected with lenti-miR-control, lenti-miR-146a-5p, lenti-miR-146a-5p mock, or lenti-miR-146a-5p EGFR-OE were evaluated by a Transwell assay (scale bars = 25 μm). **c** and **d** Migration and invasion of DU145 cells transfected with lenti-miR-control, lenti-miR-146a-5p, lenti-miR-146a-5p mock, or lenti-miR-146a-5p EGFR-OE were determined using a Transwell assay (scale bars = 25 μm). **e** After transfection with lenti-miR-control, lenti-miR-146a-5p, lenti-miR-146a-5p mock, or lenti-miR-146a-5p EGFR-OE, the protein level of E-cadherin, Vimentin, EGFR, ERK, p-ERK in PCa cells were evaluated using Western blot analysis. **f** Immunohistochemistry analysis showing the expression of p-ERK in lung metastasis among three groups (scale bars = 12.5 μm). **g** Representative Immunohistochemical staining for E-cadherin, Vimentin, EGFR and p-ERK in androgen deprived prostate tissues (Castration) and the control PCa tissues (NC) (*n* = 8). Data are shown as mean ± SD representing triplicate measurements. (Student’s t-test, * *P* < .05, **P < .01, ***P < .001, ****P < .0001)

To further study whether miR-146a-5p exerted its effects by suppressing EGFR expression in PCa cells, we restored EGFR in PCa cells co-transfected with lenti-miR-146a-5p. Transwell assays showed that EGFR overexpression (OE) reversed the effects of miR-146a-5p in both LNcaP and DU145 (Figs. 8a-d).

Interestingly, miR-146a-5p also downregulated the protein level of p-ERK1/2 but no effect on total ERK1/2 protein. Meanwhile, Upregulating EGFR expression ectopically counteracted the effects of miR-146a-5p on the protein expression of p-ERK (Fig. 8e). Furthermore, immunohistochemical staining showed that the expression level of p-ERK was elevated in lung metastasis of ETOH-treated CAF Exo group than that of DHT-treated CAF Exo group (Fig. 8f). Significantly, we found that the expression of E-cadherin was decreased and the protein level of Vimentin, EGFR and p-ERK were enhanced in androgen deprived prostate tissues (Fig. 8g).

**Fig. 9:**
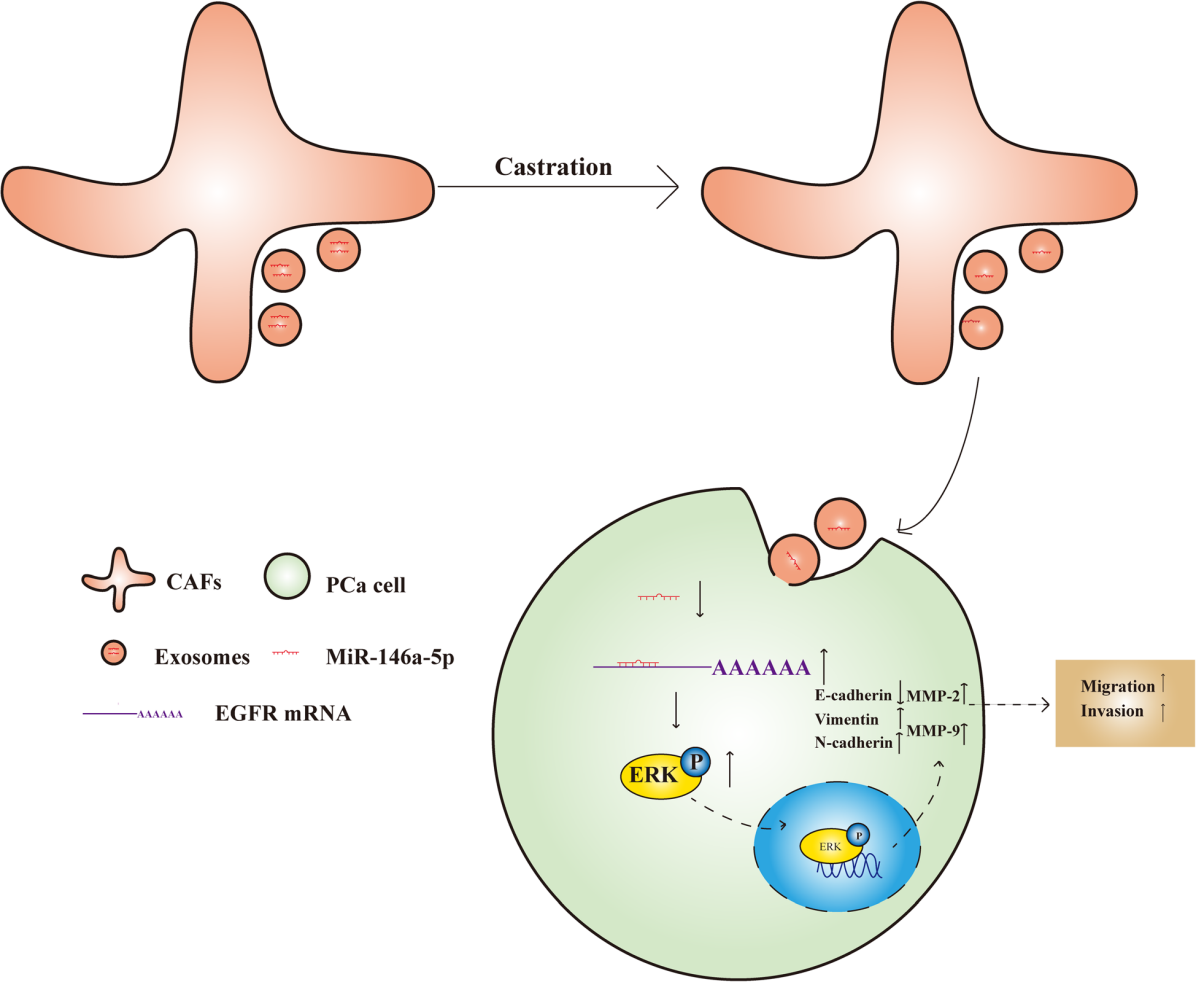
Schematic representation of the proposed mechanisms

Collectively, these results indicated that loss of exosomal miR-146a-5p promotes EMT and cell migration and invasion in PCa cells through activating EGFR/ERK pathway (Fig. 9).

## Discussion

Androgen deprivation therapy is the gold standard of care for advanced prostate cancer, but the development to castration-resistant prostate cancer is inevitable, ultimately leads to treatment failure and death. Importantly, recent studies have demonstrated that CAFs, as key components of the tumour microenvironment, contributes to PCa progression and resistance to ADT [7–9]. Furthermore, mounting studies provided new evidence to the roles of CAFs-derived exosomes in cancer progression [34, 35]. Therefore, the role of exosomes derived from CAFs exposed to ADT needs to be explored. In this paper, we showed that under castration condition, ETOH-treated CAFs-derived exosomes promote EMT, migration and invasion in PCa cells compared with exosomes derived from DHT-treated CAFs both in vitro and in vivo. Moreover, we found that after treating with ETOH-treated CAFs-derived exosomes, the expression of miR-146a-5p in cancer cells was markedly decreased than that of cells treated with DHT-treated CAFs-derived exosomes. In addition, our results suggested that overexpression of miR-146a-5p could impair the migration and invasion of PCa cells via targeting EGFR/ERK pathway. Our results confirmed that the downregulation of miR-146a-5p in exosomes derived from CAFs after ADT contributes to the metastasis of PCa cells. Besides, this study suggested that increasing the transfer of miR-146a-5p from CAFs-derived exosomes might become a new strategy to combine with ADT for the treatment of advanced prostate cancer.

Recent data showed that exosomes derived from CAFs have an important role in promoting therapy resistance in many cancer cells. Richards et al. proved that CAFs exposed to gemcitabine therapy significantly upregulate the release of exosomes that enhanced the survival of pancreatic cancer cells [36]. Qin et al. also demonstrated that in head and neck cancer (HNC), CAFs are innately chemo-resistant and compared with exosomes of CAFs, cisplatin-treated CAFs-derived exosomes dramatically promoted the proliferation and chemoresistance of HNC cells [19]. By analogy, we hypothesized that exosomes derived from CAFs with or without ADT may have different roles in regulating castration resistance or metastatic progression of PCa cells. Interestingly, we found that compared with DHT-treated CAFs-derived exosomes, ETOH-treated CAFs-derived exosomes significantly promoted the migration and invasion ability of PCa cells after ADT both in vitro and in vivo. Mechanistically, compared with DHT-treated CAFs-derived exosomes, ETOH-treated CAFs-derived exosomes activated EMT process in PCa cells. Next, we assessed the effect of ADT on exosome release in CAFs. Intriguingly, upon DHT treatment, the size and concentration of exosomes derived from CAFs have no significant difference (Fig. 1d). This finding implied that the component of exosomes derived from CAFs after ADT had changed.

MiRNAs are a type of short non-coding RNAs and are enriched in exosomes released from CAFs. Mounting evidence indicated that CAFs-derived exosomal miR-196a and miR-21 involved in therapy resistance in cancer cells [19, 20, 37].

Our results indicated that miR-146a-5p could inhibit EMT, cell migration and invasion of PCa cells in vitro. Furthermore, the results confirmed that EGFR is a direct target of miR-146a-5p in PCa cells, which was in accordance with a previous study [38]. Significantly, CAFs-derived exosomal miR-146a-5p negatively regulated the expression of EGFR in PCa cells both in vitro and in vivo, which suggested that EGFR is also a direct target of exosomal miR-146a-5p in PCa cells. Interestingly, the rescue experiment showed that exosomal miR-146a-5p exhibits its functions by inhibiting EGFR/ERK pathway in PCa cells. Our results and previous studies provide evidence for CAFs-derived exosomal miR-146a-5p as a therapeutic target for PCa.

Although there are important discoveries revealed by this study, our study still has some limitations. First, the underlying mechanism of the reduction of miR-146a-5p in exosomes derived from CAFs after ADT have not been elucidated. Intriguingly, it has been reported that AR signalling may modulate miR-146a-5p via binding to the androgen-response-elements 2 (ARE2) located on the promoter region of miR-146a-5p in hepatocellular carcinoma [39]. The mechanisms underlying the effect of AR signalling in CAFs on the level of miR-146a-5p in exosomes needed to be further investigated. Second, although we identified miR-146a-5p as the principal molecule that mediated functions of ETOH-treated CAFs-derived exosomes, other 10 significantly changed miRNAs may be involved in this process, we would investigate this in future study. Third, we could not validate the miR-146a-5p-EGFR/ERK signalling axis in metastatic clinical samples because of the availability of the metastasis foci after ADT. Therefore, the clinical significance of the results should be verified further.

## Conclusions

The present study demonstrated that exosomes from CAFs after ADT contributes to metastasis of prostate cancer. Mechanistically, loss of exosomal miR-146a-5p from CAFs promotes EMT, migration and invasion of PCa cells through EGFR/ERK signalling pathway. These findings represent a new important molecular mechanism of metastasis in prostate cancer after hormone therapy. Moreover, it suggests that increasing the exosomal transfer of miR-146a-5p from CAFs may present a new strategy to inhibit metastasis for PCa patients receiving ADT.

## Supplementary Information

**Additional file 1: Figure S1.** Characteristics of primary CAFs and CAF cell line. a: Immunofluorescence staining for α-SMA and Vimentin of primary CAFs and hTERT PF179T CAF (scale bars = 25 μm). b: The protein level of AR was detected in all CAFs by western blot and LNcaP was performed as a positive control. Data are shown as mean ± SD representing triplicate measurements. (Student’s t-test, * *P* < .05, **P < .01.).

**Additional file 2: Figure S2.** Exosomes from CAFs after ADT did not affect the proliferation of PCa cells in vitro. a and b: Cell growth rate of LNCaP and DU145 was evaluated by CCK-8 assays. Before that, cells were co-cultured with different exosomes (25 μg/mL) for 2 days under castration condition. c and d: The effect of different exosomes on the proliferation of both LNCaP and DU145 cells was assessed by EdU assays (scale bars = 25 μm). Data are shown as mean ± SD representing triplicate measurements. (Student’s t-test, ns means no significant).

**Additional file 3: Figure S3.** Exosomes from CAFs after ADT enhance the activities of MMP-2 and MMP-9 in PCa cells in vitro. a and d: Representative zymographic gels from cell culture supernatants of LNcaP and DU145 cells incubated with CAFs-derived exosomes. b, c, e and f: Statistic analysis of MMP-2 and MMP-9 activities in a and d. Data are shown as mean ± SD representing triplicate measurements. (Student’s t-test, **P < .01, ***P < .001, ****P < .0001.).

**Additional file 4: Figure S4.** The characteristics of the patients from whom CAFs were cultured.

**Additional file 5: Figure S5**. The positive rate of red signals in DU145 co-cultured with CAFs-derived exosomes. Data are shown as mean ± SD representing triplicate measurements. (Student’s t-test, * *P* < .05, **P < .01, ***P < .001, ****P < .0001.).

**Additional file 6: Figure S6.** miR-146a-5p overexpression reversed the effect of ETOH-treated CAFs-derived exosomes on the activities of MMP-2 and MMP-9 in LNcaP and DU145. a and d: LNcaP and DU145 cells were co-cultured with CAFs-derived exosomes and transfected with miR-NC or miR-146a-5p mimics for 48 h, and gelatin zymography analysed the activities of MMP-2 and MMP-9. b, c, e and f: Statistic analysis of MMP-2 and MMP-9 activities in a and d. Data are shown as mean ± SD representing triplicate measurements. (Student’s t-test, **P < .01, ***P < .001, ****P < .0001.).

**Additional file 7: Figure S7.** miR-146a-5p inhibits the activities of MMP-2 and MMP-9 in LNcaP and DU145. a and d: LNcaP and DU145 cells were transfected with miR-NC or miR-146a-5p mimics for 48 h, and gelatin zymography analysed the activities of MMP-2 and MMP-9. b, c, e and f: Statistic analysis of MMP-2 and MMP-9 activities in a and d. Data are shown as mean ± SD representing triplicate measurements. (Student’s t-test, **P < .01, ***P < .001).

**Additional file 8: Table S1**. Sequences of primers for PCR and reverse transcript.

## Data Availability

The datasets generated during the current study are available in the GEO repository (GSE154215). Other data or materials can be given by contacting us.

## Abbreviations

AR: Androgen receptor
PCa: Prostate cancer
CAFs: Cancer-associated fibroblasts
CCK-8: Cell counting kit-8
CSFBS: Charcoal-stripped fetal bovine serum
CRPC: Castration-resistant prostate cancer
DHT: Dihydrotestosterone
ETOH: Ethanol
EMT: Epithelial-to-mesenchymal transition
FBS: Fetal bovine serum
IVIS: In vivo imaging system
qRT-PCR: quantitative real-time polymerase chain reaction
