# Supplementary figures and images for: Loss of exosomal miR-146a-5p from cancer-associated fibroblasts after androgen deprivation therapy contributes to prostate cancer metastasis
Source: J Exp Clin Cancer Res. 2020 Dec 14;39:282. doi: 10.1186/s13046-020-01761-1 (PMC7734763; doi:10.1186/s13046-020-01761-1)

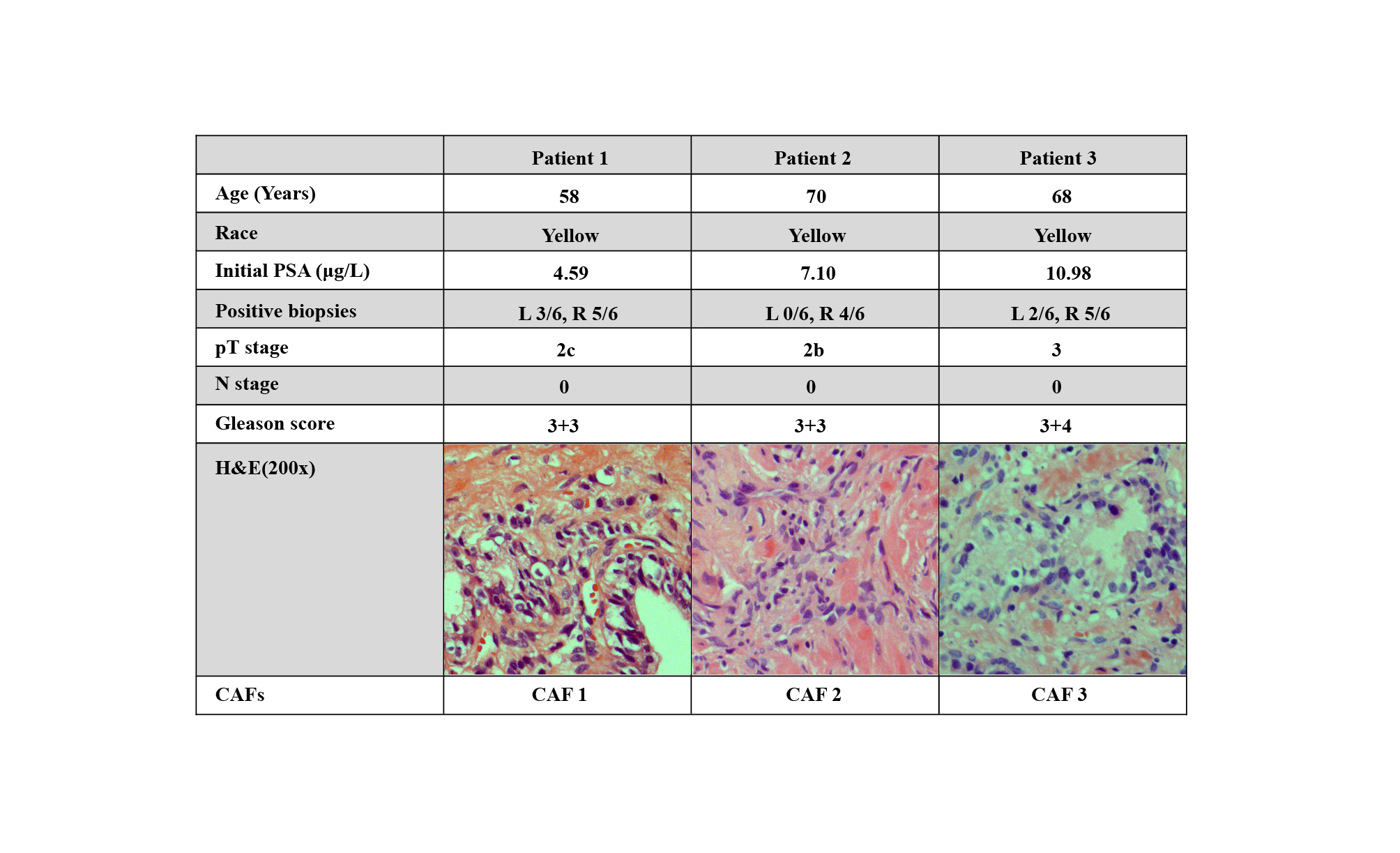

Supplement: Supplementary file 1 — Additional file 1: Figure S1. Characteristics of primary CAFs and CAF cell line. a: Immunofluorescence staining for α-SMA and Vimentin of primary CAFs and hTERT PF179T CAF (scale bars = 25 μm). b: The protein level of AR was detected in all CAFs by western blot and LNcaP was performed as a positive control. Data are shown as mean ± SD representing triplicate measurements. (Student’s t-test, * P < .05, **P < .01.). [file 13046_2020_1761_MOESM1_ESM.tif]

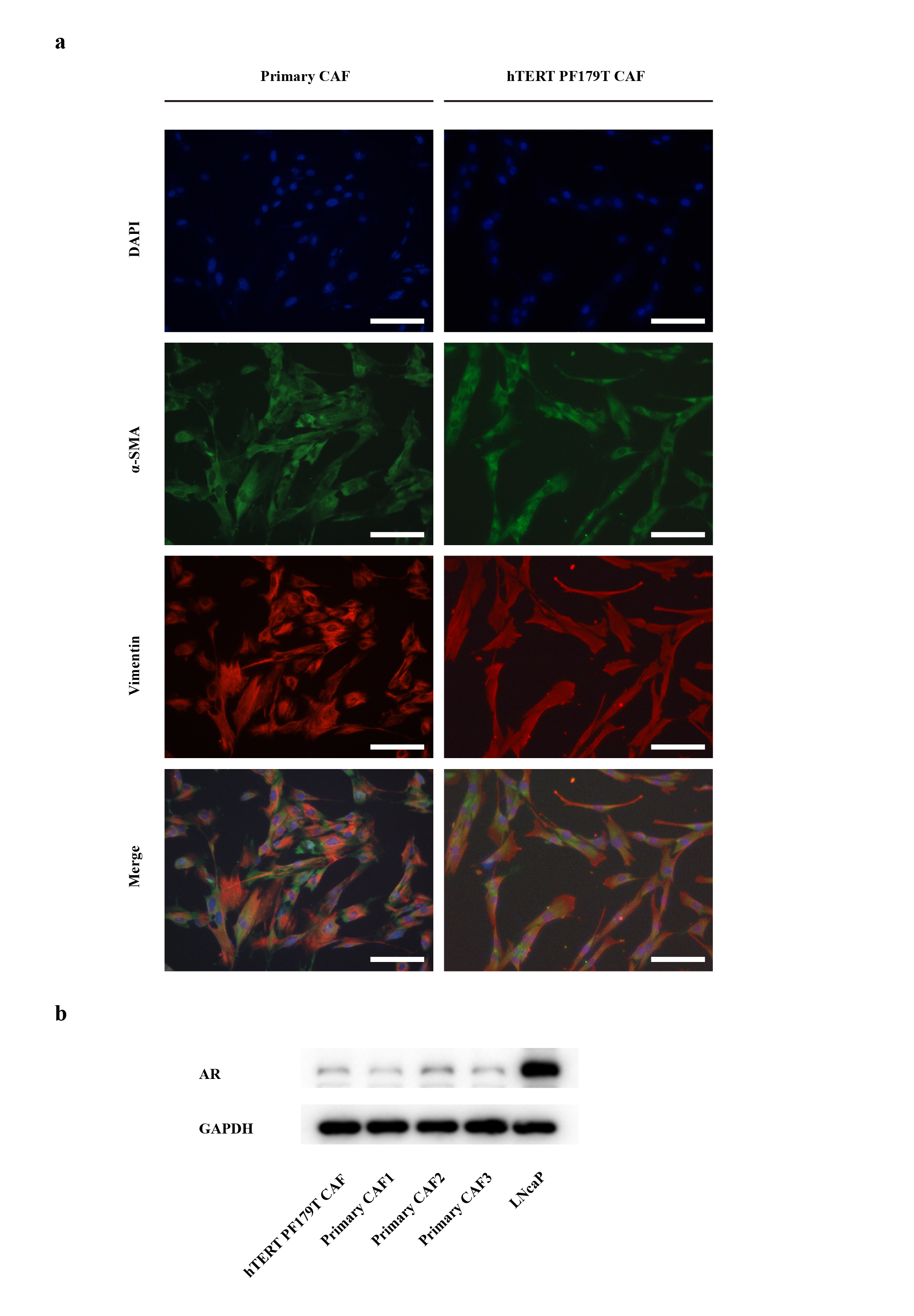

Supplement: Supplementary file 2 — Additional file 2: Figure S2. Exosomes from CAFs after ADT did not affect the proliferation of PCa cells in vitro. a and b: Cell growth rate of LNCaP and DU145 was evaluated by CCK-8 assays. Before that, cells were co-cultured with different exosomes (25 μg/mL) for 2 days under castration condition. c and d: The effect of different exosomes on the proliferation of both LNCaP and DU145 cells was assessed by EdU assays (scale bars = 25 μm). Data are shown as mean ± SD representing triplicate measurements. (Student’s t-test, ns means no significant). [file 13046_2020_1761_MOESM2_ESM.tif]

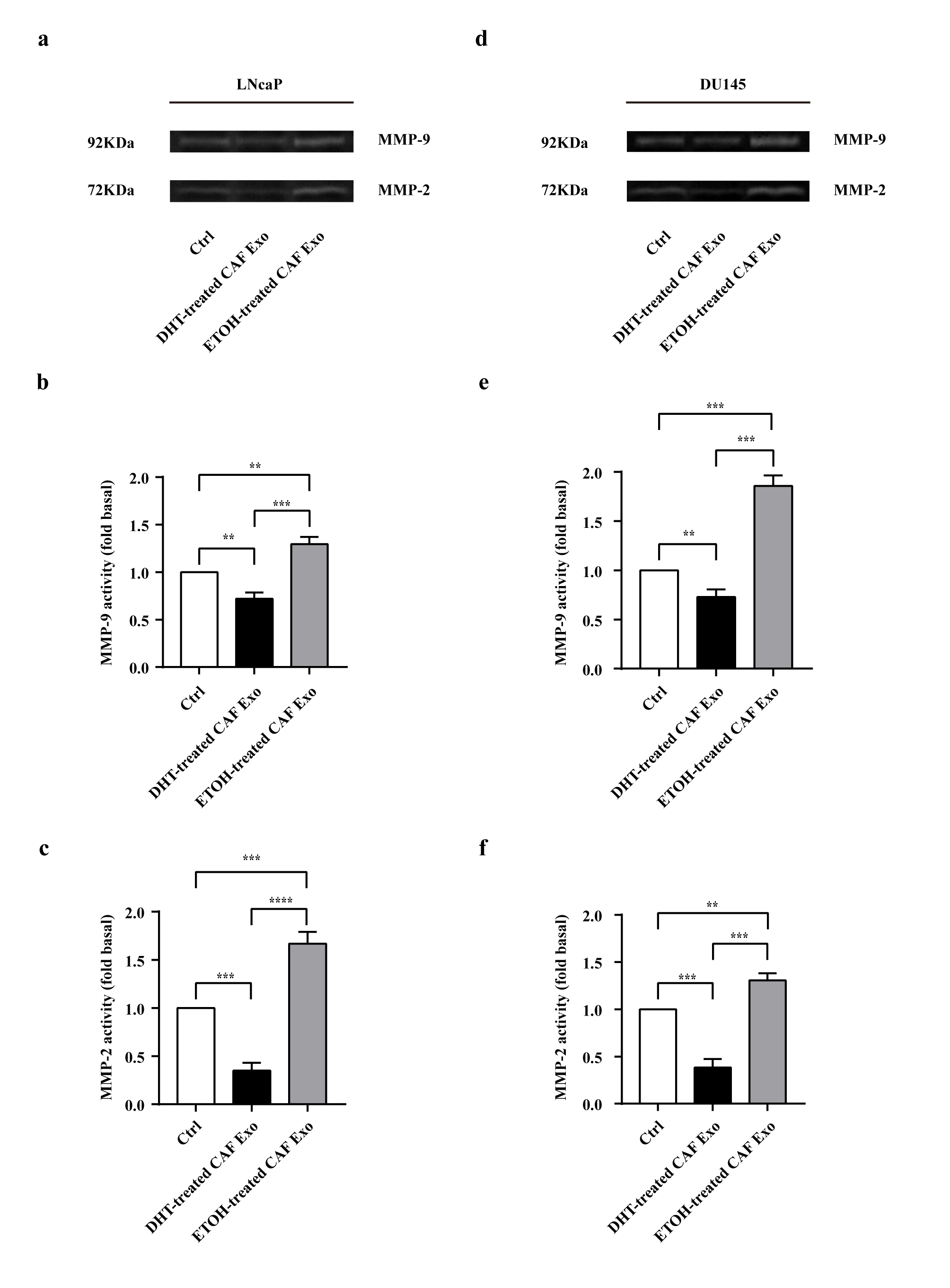

Supplement: Supplementary file 3 — Additional file 3: Figure S3. Exosomes from CAFs after ADT enhance the activities of MMP-2 and MMP-9 in PCa cells in vitro. a and d: Representative zymographic gels from cell culture supernatants of LNcaP and DU145 cells incubated with CAFs-derived exosomes. b, c, e and f: Statistic analysis of MMP-2 and MMP-9 activities in a and d. Data are shown as mean ± SD representing triplicate measurements. (Student’s t-test, **P < .01, ***P < .001, ****P < .0001.). [file 13046_2020_1761_MOESM3_ESM.tif]

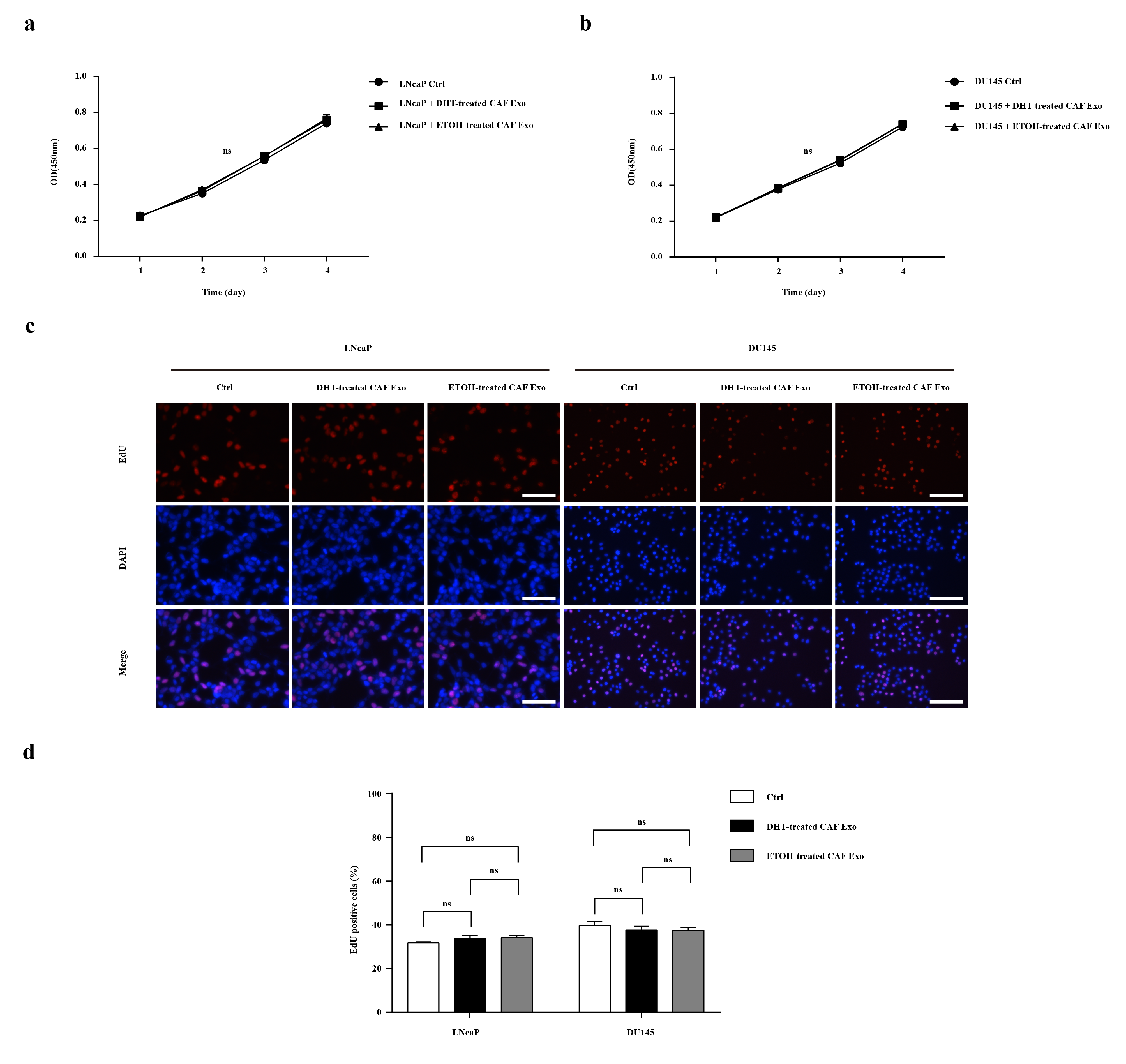

Supplement: Supplementary file 4 — Additional file 4: Figure S4. The characteristics of the patients from whom CAFs were cultured. [file 13046_2020_1761_MOESM4_ESM.tif]

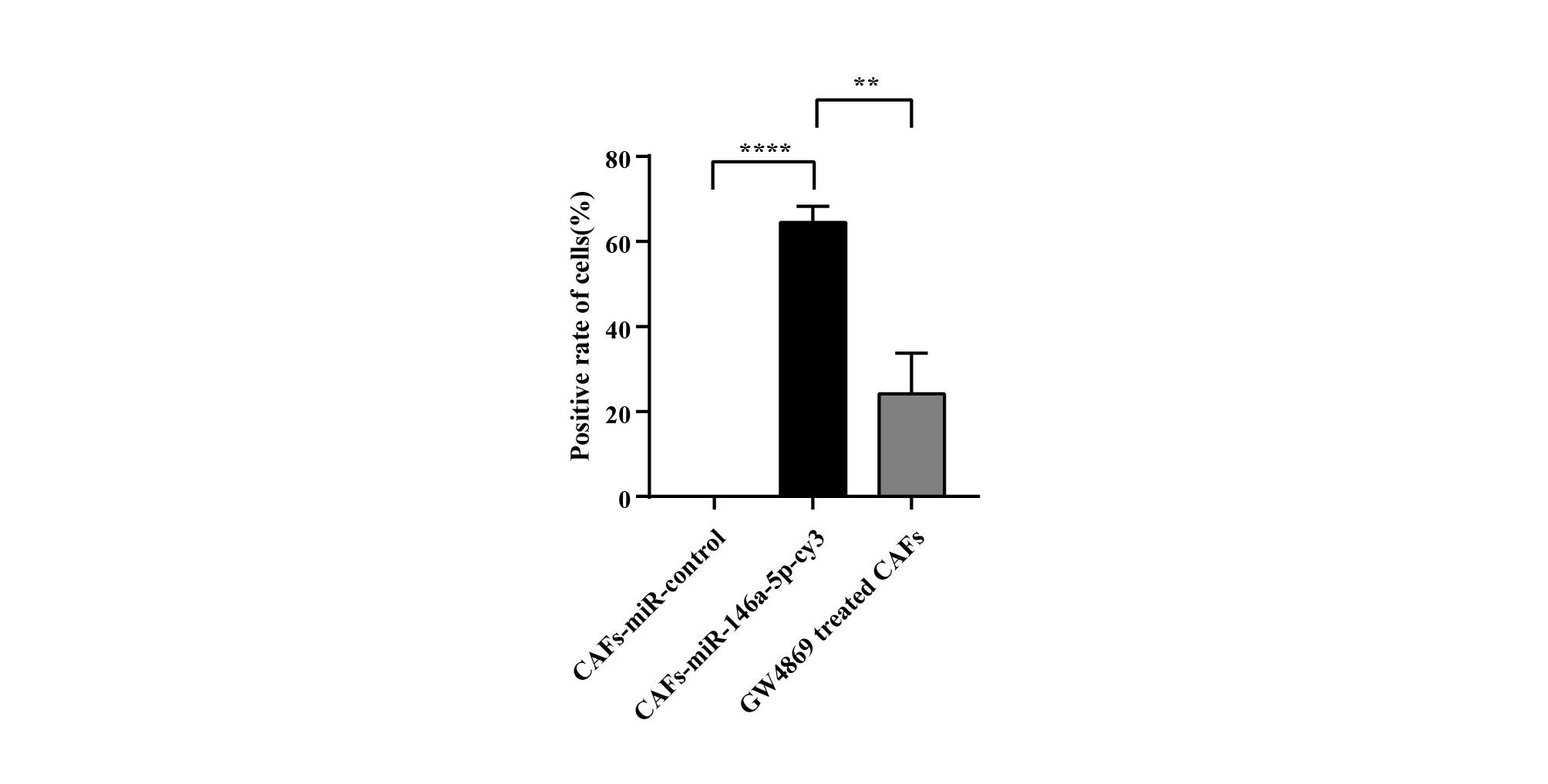

Supplement: Supplementary file 5 — Additional file 5: Figure S5. The positive rate of red signals in DU145 co-cultured with CAFs-derived exosomes. Data are shown as mean ± SD representing triplicate measurements. (Student’s t-test, * P < .05, **P < .01, ***P < .001, ****P < .0001.). [file 13046_2020_1761_MOESM5_ESM.tif]

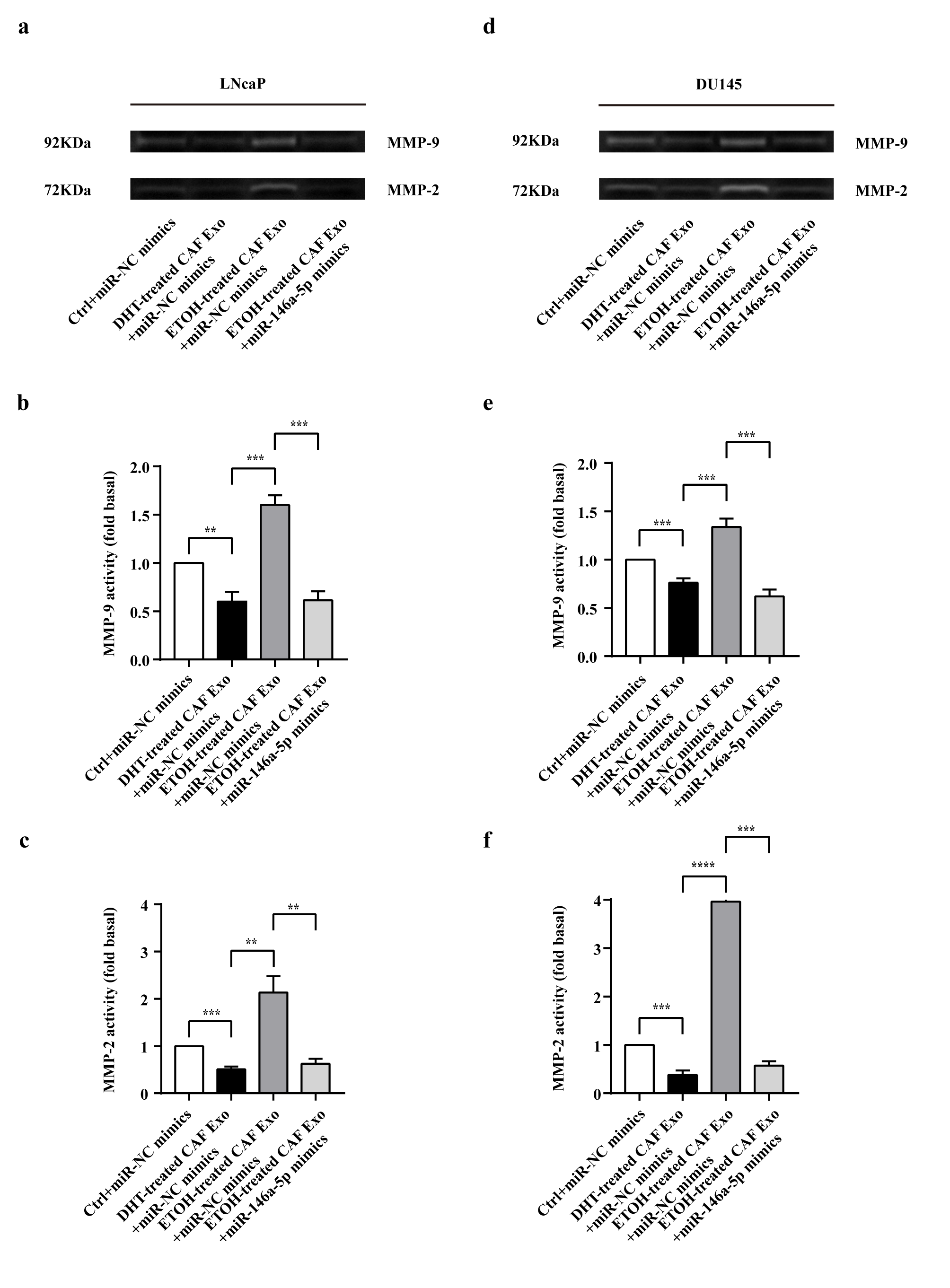

Supplement: Supplementary file 6 — Additional file 6: Figure S6. miR-146a-5p overexpression reversed the effect of ETOH-treated CAFs-derived exosomes on the activities of MMP-2 and MMP-9 in LNcaP and DU145. a and d: LNcaP and DU145 cells were co-cultured with CAFs-derived exosomes and transfected with miR-NC or miR-146a-5p mimics for 48 h, and gelatin zymography analysed the activities of MMP-2 and MMP-9. b, c, e and f: Statistic analysis of MMP-2 and MMP-9 activities in a and d. Data are shown as mean ± SD representing triplicate measurements. (Student’s t-test, **P < .01, ***P < .001, ****P < .0001.). [file 13046_2020_1761_MOESM6_ESM.tif]

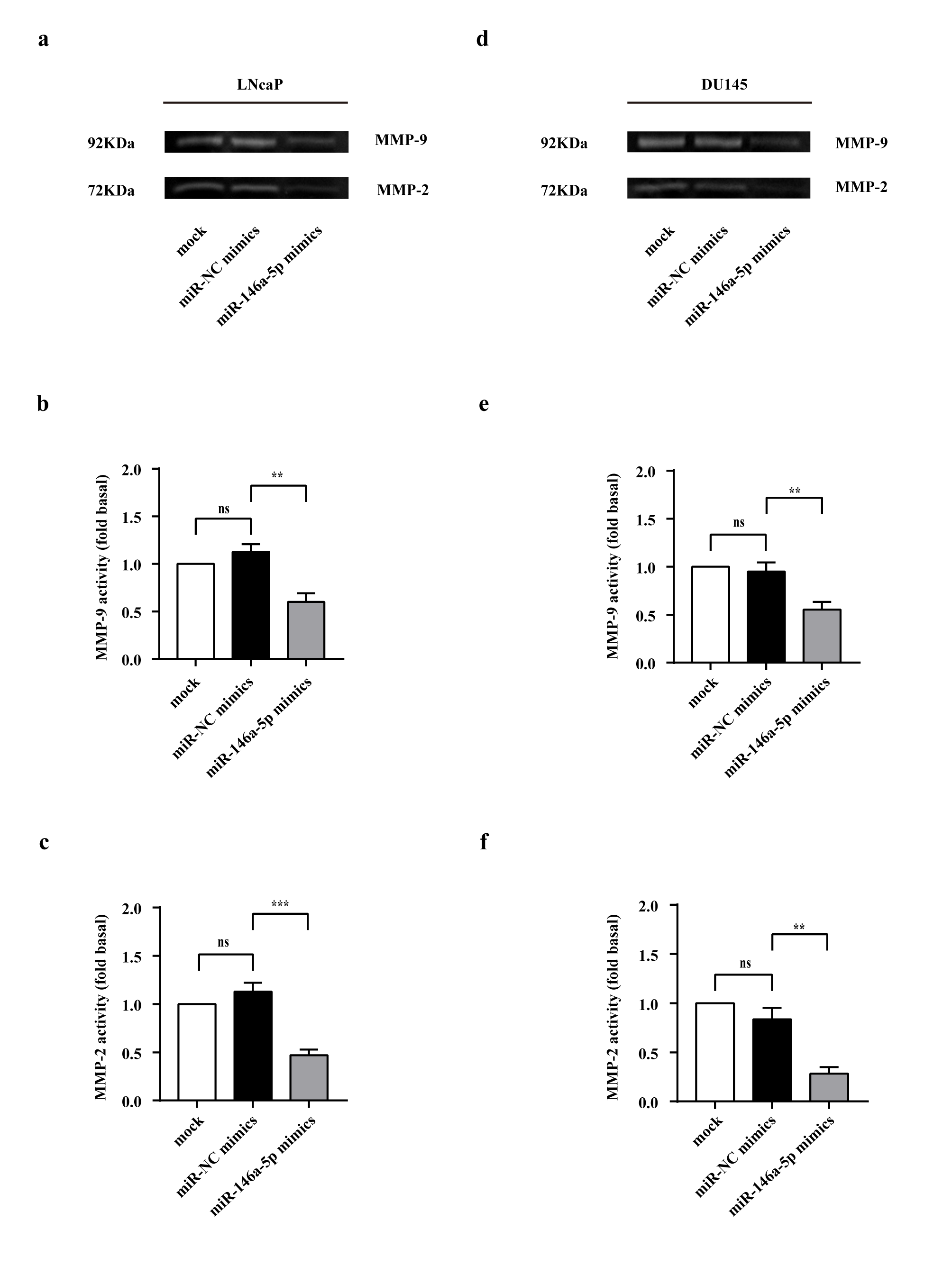

Supplement: Supplementary file 7 — Additional file 7: Figure S7. miR-146a-5p inhibits the activities of MMP-2 and MMP-9 in LNcaP and DU145. a and d: LNcaP and DU145 cells were transfected with miR-NC or miR-146a-5p mimics for 48 h, and gelatin zymography analysed the activities of MMP-2 and MMP-9. b, c, e and f: Statistic analysis of MMP-2 and MMP-9 activities in a and d. Data are shown as mean ± SD representing triplicate measurements. (Student’s t-test, **P < .01, ***P < .001). [file 13046_2020_1761_MOESM7_ESM.tif]
